# Genetic adaptations to SIV across chimpanzee populations

**DOI:** 10.1371/journal.pgen.1010337

**Published:** 2022-08-25

**Authors:** Harvinder Pawar, Harrison J. Ostridge, Joshua M. Schmidt, Aida M. Andrés

**Affiliations:** 1 UCL Genetics Institute, Department of Genetics, Evolution and Environment, University College London, London, United Kingdom; 2 Department of Ecology and Evolution, School of Biological Sciences, University of Adelaide, Adelaide, Australia; University of Pennsylvania, UNITED STATES

## Abstract

Central and eastern chimpanzees are infected with Simian Immunodeficiency Virus (SIV) in the wild, typically without developing acute immunodeficiency. Yet the recent zoonotic transmission of chimpanzee SIV to humans, which were naïve to the virus, gave rise to the Human Immunodeficiency Virus (HIV), which causes AIDS and is responsible for one of the deadliest pandemics in human history. Chimpanzees have likely been infected with SIV for tens of thousands of years and have likely evolved to reduce its pathogenicity, becoming semi-natural hosts that largely tolerate the virus. In support of this view, central and eastern chimpanzees show evidence of positive selection in genes involved in SIV/HIV cell entry and immune response to SIV, respectively. We hypothesise that the population first infected by SIV would have experienced the strongest selective pressure to control the lethal potential of zoonotic SIV, and that population genetics will reveal those first critical adaptations. With that aim we used population genetics to investigate signatures of positive selection in the common ancestor of central-eastern chimpanzees. The genes with signatures of positive selection in the ancestral population are significantly enriched in SIV-related genes, especially those involved in the immune response to SIV and those encoding for host genes that physically interact with SIV/HIV (VIPs). This supports a scenario where SIV first infected the central-eastern ancestor and where this population was under strong pressure to adapt to zoonotic SIV. Interestingly, integrating these genes with candidates of positive selection in the two infected subspecies reveals novel patterns of adaptation to SIV. Specifically, we observe evidence of positive selection in numerous steps of the biological pathway responsible for T-helper cell differentiation, including *CD4* and multiple genes that SIV/HIV use to infect and control host cells. This pathway is active only in CD4+ cells which SIV/HIV infects, and it plays a crucial role in shaping the immune response so it can efficiently control the virus. Our results confirm the importance of SIV as a selective factor, identify specific genetic changes that may have allowed our closest living relatives to reduce SIV’s pathogenicity, and demonstrate the potential of population genomics to reveal the evolutionary mechanisms used by naïve hosts to reduce the pathogenicity of zoonotic pathogens.

## Author summary

Chimpanzees are at the origin of HIV-1, a virus that generates an incurable disease and that generated a pandemic that has claimed 35 million lives. Chimpanzees have evolved to control the pathogenicity of the virus, which does not typically develop into AIDS in the same way as in humans. Identifying the genetic adaptations responsible for this process provides critical knowledge about SIV and HIV. Our analysis of chimpanzee genetic adaptations identified specific genes and molecular pathways involved in adaptation to SIV, providing important insights into the mechanisms that likely allowed our closest living relatives to control SIV/HIV. Further, we establish SIV as a strong and recurrent selective pressure in central and eastern chimpanzees, two important subspecies of large mammals that are currently endangered.

## Introduction

A typical evolutionary outcome of pathogen infections is the development of host immunity or tolerance to an infectious agent that was initially highly pathogenic, and that remains so in naïve species. Host genetic adaptations underlie the sometimes striking differences in infection outcome in different species infected by the same virus. The lentivirus Simian Immunodeficiency Virus (SIV) provides a fascinating example of this process. Most African primates, the great apes being notable exceptions, have their own endemic SIV strain for which they are considered natural hosts [1]. When natural hosts (e.g. vervet monkeys) are exposed to their endemic SIV, they have a proportionate immune activation that preserves CD4+ T cell counts and wider immune function, resulting in asymptomatic outcomes and no significant reduction in lifespan despite viral replication [2–4]. In contrast, species that are naïve to SIV, such as non-African primates, are unable to resolve the acute phase of infection and instead generate chronic immune activation, CD4+ T cell depletion and eventual immunodeficiency [1,5–7]. The course of these infections and the development of clinical disease is almost identical to that of humans infected with HIV, which are also a naïve species and which, without treatment, progress to acquired immunodeficiency syndrome (AIDS).

Humans have received multiple zoonotic transmissions of SIV. The most deadly, which introduced HIV-1 group M responsible for the AIDS pandemic, originated when the SIV of a central chimpanzee (*Pan troglodytes troglodytes*) jumped into humans in the early twentieth century [8,9]. HIV-1 is thus genetically very similar to chimpanzee SIV (SIVcpz) [8]. Also, the chimpanzee genome has a high sequence identity to the human genome [10] and the two species share most aspects of their physiology. Therefore, the mechanisms used by chimpanzees to limit the pathogenicity of SIV may be informative about potential mechanisms to control HIV.

Chimpanzees have traditionally been considered natural SIV hosts, because despite infections in zoos and laboratories, infected chimpanzees rarely progress to AIDS-like symptoms [11–13]. Further, SIV-infected chimpanzees show proportionate patterns of immune activation, similar to those observed in natural hosts, such as vervet monkeys [14]. Yet, recent observations have suggested that chimpanzees are not true natural hosts, instead lying between natural and symptomatic SIV hosts. Disease symptoms such as CD4+ T-cell depletion and thrombocytopenia have been observed in infected chimpanzees and associated with reduced fitness, particularly in the wild [14–16]. Specifically, Keele *et al*., observed in habituated wild eastern chimpanzees that SIV infection increased mortality risk and infected females had fewer offspring and higher infant mortality rates than uninfected females [15]. Similarly, SIV infection has been associated with population decline in the Kalande community of eastern chimpanzees [17]. It should be noted that studies investigating the fitness effects of SIVcpz in the wild have been restricted to small sample sizes from few habituated populations due to the inherent challenges associated with studying wild populations of endangered large mammals. Nevertheless, evidence suggests that chimpanzees can be considered ‘semi-natural’ hosts, as the virus lacks the dramatic pathogenicity seen in naïve species like humans yet likely has non-negligible health consequences and fitness effects. This is perhaps expected if chimpanzees have already acquired adaptations that control the high initial pathogenicity of SIV but have not yet acquired adaptations that fully control the effects of the infection.

**Fig 1:**
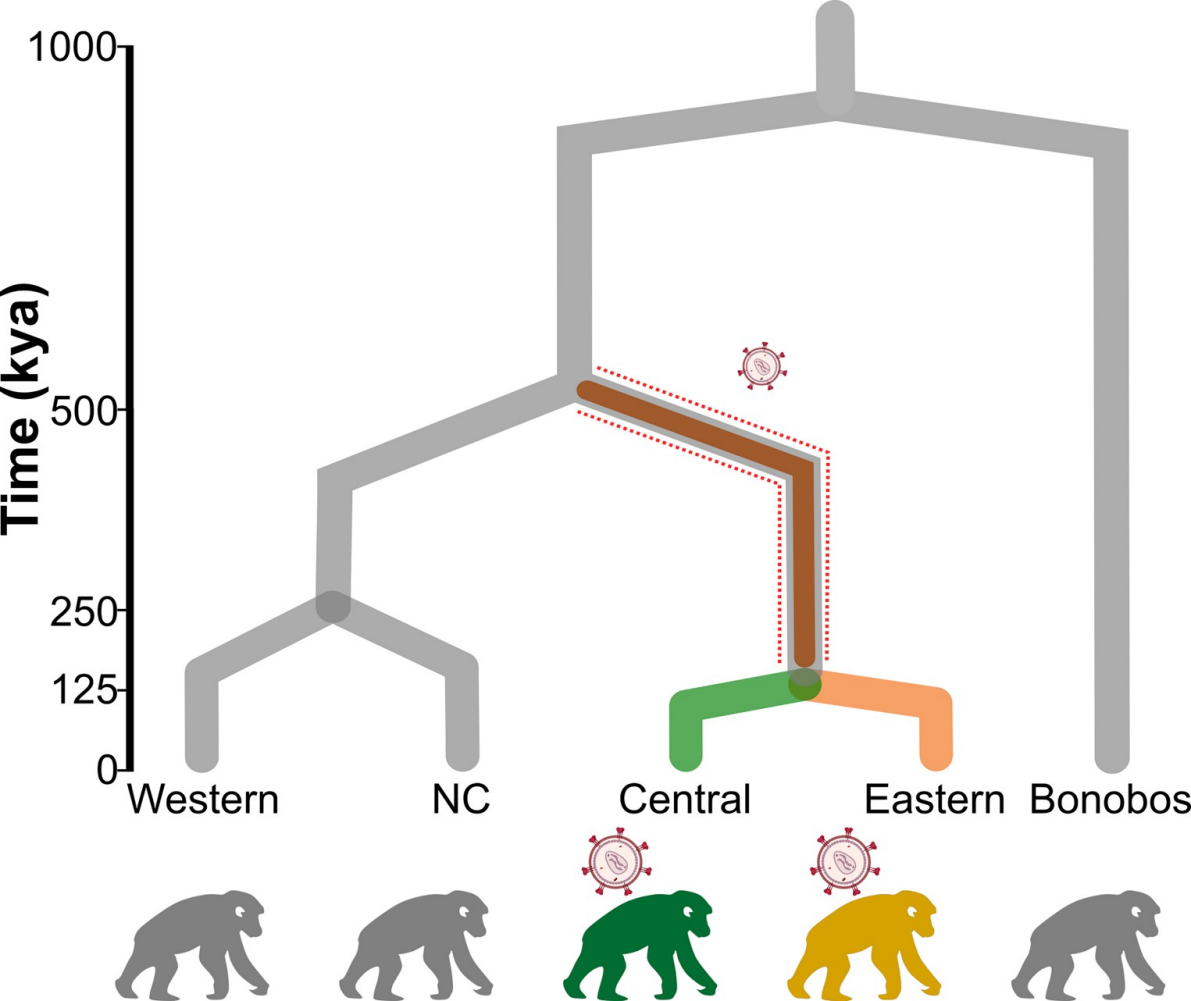
Phylogeny of chimpanzee subspecies and bonobos with the distribution of natural SIV infection. The dotted red line indicates the central-eastern ancestor where the first SIV infection possibly occurred, for which we investigate positive selection.

Interestingly, of the four genetically and geographically distinct subspecies of chimpanzee, natural SIV infection is found only in central (*P*. *t*. *troglodytes*) and eastern (*P*. *t*. *schweinfurthii*) chimpanzees, who are most closely related to each other [18] (Fig 1). SIV infection has not been detected in the wild in the second clade of chimpanzees, which includes the western (*P*. *t*. *verus*) and Nigeria-Cameroon (*P*. *t*. *ellioti*) subspecies [8,18,19]. The uneven distribution of SIV among chimpanzee subspecies has sparked much interest in the origin of the virus in the *Pan* lineage.

We know that SIVcpz is the result of zoonotic transmission to chimpanzees of at least two African monkey SIV strains (SIV from the red-capped mangabey and the ancestor of the SIVs currently infecting mona, moustached and greater spot-nosed monkeys), which then recombined and generated SIVcpz, a mosaic lineage able to infect and transmit in chimpanzees [20,21]. Given this complex origin, it is most likely that SIVcpz arose only once. More than one scenario is theoretically compatible with the distribution of SIVcpz in nature. Zoonotic transmission to the common ancestor of all chimpanzees and subsequent clearance in western and Nigeria-Cameroon is highly unlikely given the current SIVcpz distribution [11,22], as it would require the virus to be lost in two subspecies. Zoonotic transmission into the central-eastern clade is more likely, either through transmission to the common ancestor of central and eastern chimpanzees or through transmission to one subspecies (e.g. central) with subsequent transfer to the other subspecies (e.g. eastern). A reliable estimate of the time of the most recent common ancestor of central and eastern SIVcpz could be informative about the likelihood of these two scenarios, but the imprecision of phylogenetic dating of SIVcpz, which has been highly variable [23,24] due to the difficulties associated with dating deep times scales for SIV [24], complicate such inferences. The phylogeny of SIVcpz separates two monophyletic sister clades, one containing all SIVcpzPtt sequences (the virus infecting central chimpanzees) and one containing all SIVcpzPts sequences (the virus infecting eastern chimpanzees) [11,25], which could be compatible with both scenarios. SIVcpzPts lineages do not fall within the diversity of SIVcpzPtt, or vice versa [25], as we would expect if one subspecies (e.g. centrals) was originally infected and recently infected the other subspecies (e.g. easterns). However, if SIVcpz was laterally transmitted sufficiently long ago, lineage extinction could generate reciprocal monophylogeny in this scenario too. So while the two scenarios are not equally likely, distinguishing among them is difficult with current data.

SIV is a dangerous pathogen, and as such a strong selective force. Once SIV infects a species, SIV-related adaptation is likely to be pervasive and continuous. In fact even natural hosts such as vervet monkeys, which have been infected for 0.5–3 million years, show genome-wide signatures of positive selection in SIV-related genes [3,26]. It has long been thought that SIV may be a strong selective force in chimpanzees, posited to drive allele frequency change and adaptation in a few immune and SIV-related genes [27–31]. Analysing dozens of genomes, we confirmed that SIV has driven chimpanzee evolution by uncovering the presence of recent adaptations to SIV in the central and eastern subspecies [32]. Using the PBSnj statistic, we identified the SNPs with the greatest allele frequency change in each chimpanzee subspecies, which are the strongest candidate targets of subspecies-specific positive selection. Strikingly, both in central and eastern chimpanzees, these SNPs are enriched in genes related to SIV. As expected under a model of zoonotic transmission into the central-eastern clade, neither western nor Nigeria-Cameroon showed evidence of recent positive selection in SIV-related gene categories [32]. Although earlier work had reported evidence of low diversity at *CCR5*, *CXCR4* and *CX3CR1* in Nigeria-Cameroon and western, and suggested that this may be due to positive selection [30,31], it is unclear if SIV was the selective force.

The evidence of genetic adaptation in sets of SIV-related genes is clearer and easy to interpret. Identified candidate targets of recent selection in central chimpanzees are enriched in cytokine coreceptors due to signatures in *CCR3*, *CCR9* and *CXCR6* [32], which mediate HIV cell entry together with the primary receptor *CD4* [33–35] and are paralogs of the HIV coreceptors *CCR5* and *CXCR4* [36–38]. In contrast, selection targets in eastern chimpanzees are enriched in “SIV-response genes”, genes that upon SIV infection show different expression profiles in CD4+ T lymphocytes in natural host vs. naïve host species (in this case, vervet monkeys vs macaques, [26,39,40]). These SIV-response genes likely contribute to the finely tuned natural host response to SIV, which is able to control the infection and results in non-pathogenic outcomes [32]. Thus, interestingly, the two subspecies seem to have evolved differential adaptation mechanisms to control SIV.

Under a scenario of zoonotic transmission into the central-eastern ancestor, this naïve population would have been under strong pressure to adapt–actually, it would be the chimpanzee population with the strongest pressure to adapt. We thus hypothesise that under this scenario positive selection in the central-eastern ancestor would have been critical to control the lethal potential of zoonotic SIV upon a naïve chimpanzee population. Under that hypothesis, we could detect signatures of positive selection in SIV-related genes in the central-eastern ancestor. We test this hypothesis using 47 chimpanzee whole genomes. We find evidence of adaptation in genes involved in SIV biology, providing further support for SIV infection in the central-eastern ancestor. Further, candidate targets of positive selection point to diverse adaptive mechanisms, including host response to infection, viral interactions and cell entry. Combining information across populations we discover previously unknown mechanisms of adaptation to SIV. Specifically, we identify the T helper cell type-1/type-2 (Th1/Th2) differentiation pathway as a critical player that has been repeatedly targeted by positive selection at different time points during chimpanzee evolution. This reveals the potential of this functional pathway, and specific genes within the pathway, to control the pathogenicity of SIV/HIV infection.

Excitingly, population genetics tools allow us to identify the genetic adaptations that happened at this critical time during which chimpanzees evolved to reduce the pathogenicity of a potentially deadly virus.

## Results

### Signatures of positive selection in the central-eastern ancestor

We identified genomic regions showing evidence of positive selection in the central-eastern ancestor using 3P-CLR [41]. 3P-CLR tests distortions of the site frequency spectrum (SFS) due to selective sweeps, by modelling the evolutionary trajectory of alleles in a 3-population tree and comparing the likelihood of the observed SFS under contrasting hypotheses of neutrality and positive selection [41]. 3P-CLR is ideal for our purpose. First, it has high power to detect the events we target, as it was designed to identify hard sweeps in ancestral modern humans using Neanderthals as an outgroup [41], and the modern human-Neanderthal divergence [42] is on the order of the inferred split time between chimpanzee clades [43]. We established with simulations that 3P-CLR also has very high power to detect selective events in the central-eastern ancestor when Nigeria-Cameroon is used as outgroup (Fig A in S1 Appendix), with power equal or higher than that reported in humans [41] under the same scenario: strong selection and fixation of the advantageous allele. Second, 3P-CLR has high power to detect selection in the ancestral population while being largely unaffected by convergent evolution (independent selection in both central and eastern chimpanzees) that does not generate the SFS distortions that translate into high 3P-CLR likelihood ratio scores [41].

**Fig 2:**
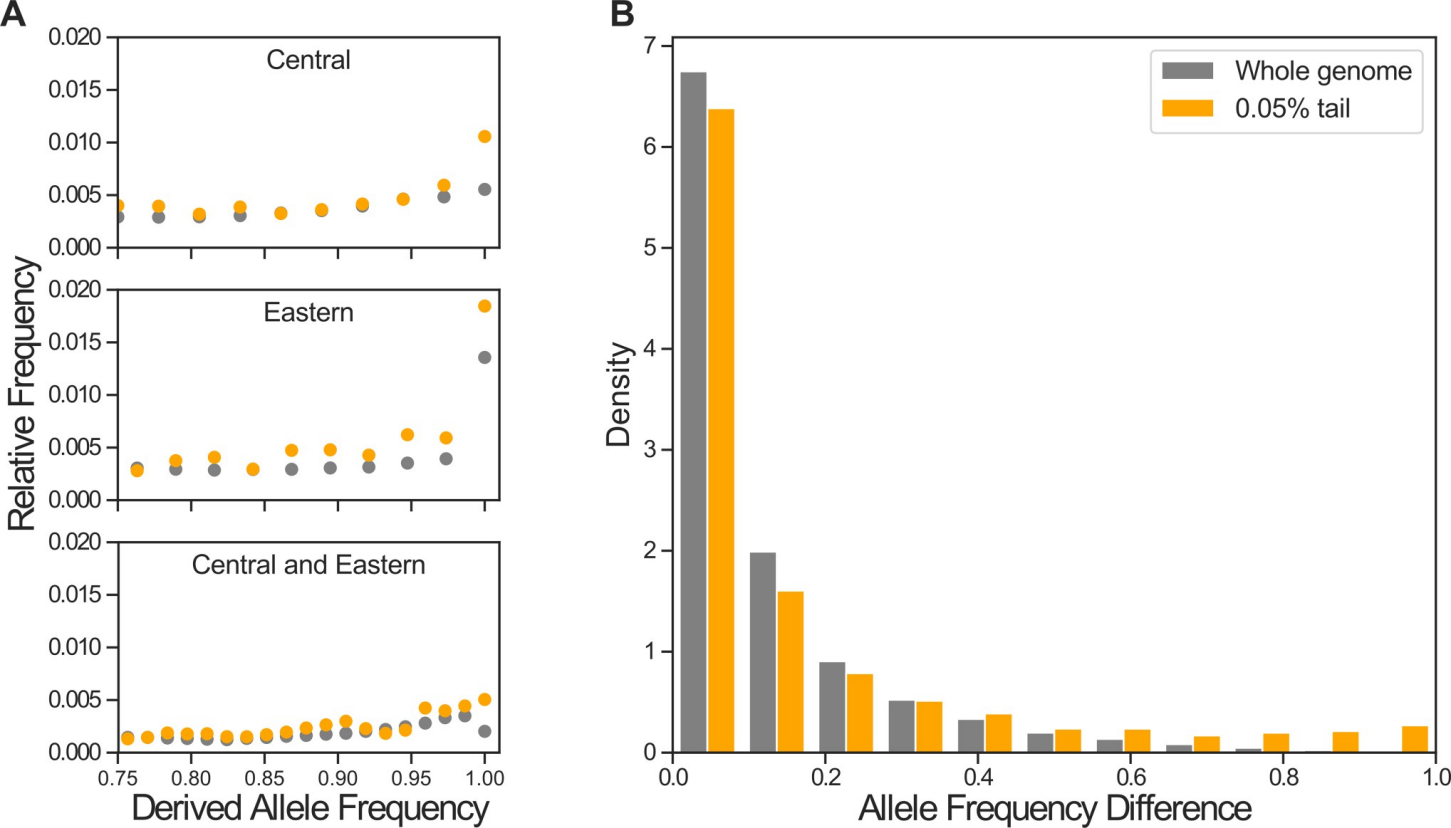
Site frequency patterns of SNPs in candidate windows. Allele frequencies of SNPs genome-wide (grey) and at the most stringent 3P-CLR tail threshold (0.05% candidates for positive selection in the central-eastern ancestor, orange). **A**: Unfolded SFS for central, eastern and central and eastern combined. The X axis is limited to focus on high-frequency derived alleles, full SFS in Fig B in S1 Appendix, SFS at all 3P-CLR tail thresholds in Fig C in S1 Appendix. **B**: Absolute DAF difference between central-eastern and Nigeria-Cameroon.

Informed by the power analysis, we applied 3P-CLR to the central–eastern–Nigeria-Cameroon 3-population tree, using the genotypes from high-coverage autosomal genomes of 47 chimpanzees (18 central, 19 eastern and 10 Nigeria-Cameroon) [43], sliding windows of size 0.25 centiMorgans (cM) and the recombination map of Auton *et al*., [44]. The windows with the highest 3P-CLR scores in the genome-wide empirical distribution have the strongest evidence of positive selection at this time depth. We thus consider candidate targets of positive selection the windows with the highest 99.5%, 99.9% and 99.95% 3P-CLR likelihood ratio scores in the genome, which correspond to the 0.5% (n = 4090), 0.1% (n = 818) and 0.05% (n = 409) tails of the empirical distribution. The candidate windows have additional signatures that, while not fully independent from the 3P-CLR signatures, are expected under positive selection in the central-eastern ancestor: they contain a marked excess of sites with high derived allele frequencies (DAF) in central and eastern (Fig 2A), and an excess of highly differentiated SNPs between the central-eastern clade and Nigeria-Cameroon (Fig 2B). While we cannot discard the presence of some false positives, these genomic regions are prime candidates to have mediated genetic adaptations in the central-eastern ancestral population.

### SIV-related selection in the central-eastern ancestor

Under the hypothesis that SIV was a strong driver of adaptive evolution in the central-eastern ancestor, genes with signatures of positive selection would fall in known SIV-related functions more often than expected by chance. We test this expectation with enrichment tests for existing SIV/HIV-related categories on the genes that overlap our candidate windows, using Gowinda [45]. Gowinda tests for overrepresentation of a given gene set in our candidate windows compared to the expectation under neutrality [45] (see Methods).

**Fig 3:**
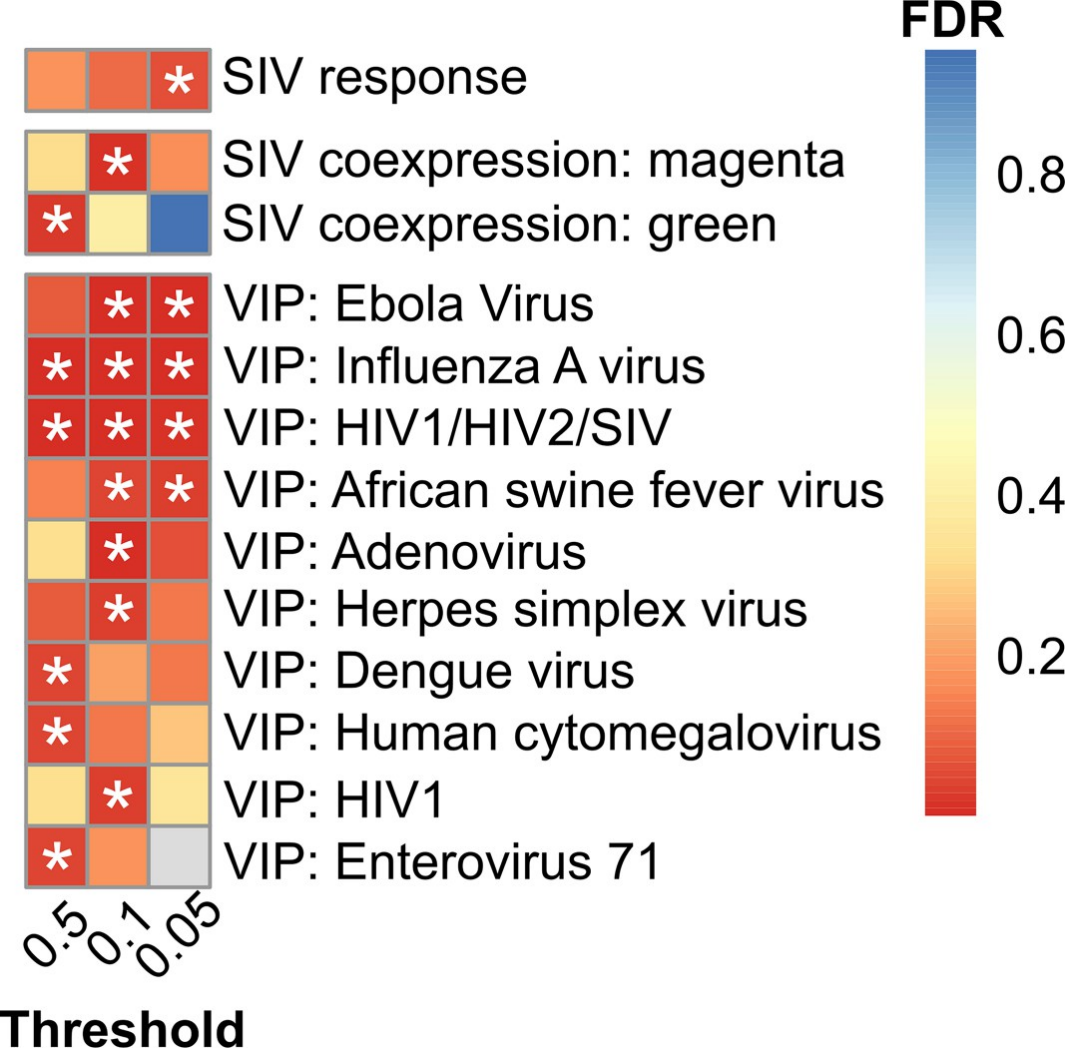
Enrichment of HIV/SIV-related and VIP categories in 3P-CLR candidate genes for the central-eastern ancestor. Columns for the three 3P-CLR quantiles, rows for each category that is significantly enriched in at least one quantile (FDR<0.05). From top to bottom: SIV response genes [39,40], SIV co-expression modules [26] and VIPs [46,47]. For the SIV-response gene set there is only one category, hence we consider p-value<0.05 as significant, following [32]. Colours represent FDR values, with grey representing categories undetected in that particular quantile. Stars mark categories with FDR < 0.05.

First we investigate selection on the immune response to SIV, by testing for an enrichment among the candidates in SIV-response genes, which in natural hosts change expression after SIV infection [39,40] and whose concerted action is thought to control SIV infection in natural hosts [39,40]. SIV-response genes are enriched in signatures of positive selection in eastern chimpanzees [32] and vervet monkeys [26], suggesting that they mediate adaptation to SIV. We find that the strongest candidate targets of positive selection in the central-eastern ancestor are modestly enriched in SIV-response genes, (0.05% candidates threshold, 11.8 expected, 18 observed, p-value = 0.043; Fig 3). This signature can be refined by exploring enrichment in the 33 modules of differentially expressed genes that co-express temporally during SIV infection, defined by Svardal *et al*., [26]. Two SIV co-expression modules are significantly enriched among candidate genes (Fig 3), both of which are defined by an acute response to SIV infection six days post-infection and exhibit strong signatures of positive selection in vervet monkeys [26]. Six days post-infection corresponds to when SIV can be first detected and when the immune response is typically initiated in natural hosts [26].

Next we investigate selection on host-virus physical protein interactions, by testing whether the candidates are enriched in genes that encode Viral Interacting Proteins (VIPs)–host proteins that physically interact with viral proteins, viral RNA or viral DNA [46,47]. HIV/SIV-interacting VIPs have not been shown to be enriched in signatures of positive selection in chimpanzees or vervet monkeys, but VIPs are clear mediators of adaptation to viruses [46,47]. Testing enrichment in all the 152 defined VIP categories, one of the strongest and most consistent enrichment signals is in VIPs that interact with HIV/SIV, across all candidate cut-offs (0.5% candidates threshold, p-value = 0.00046; 0.1% candidates threshold, p-value = 0.00014; 0.05% candidates threshold, p-value = 0.00398; FDR values given in Fig 3). Interestingly, we find similarly strong enrichment in one additional category: influenza-interacting VIPs (0.5% candidates threshold, p-value = 0.00002; 0.1% candidates threshold, p-value = 0.00010; 0.05% candidates threshold, p-value = 0.00014; FDR values given in Fig 3), in agreement with recent work in humans suggesting that RNA viruses are an important selective force in mammals [46]. Given the differences in gene set sizes between the influenza and SIV/HIV (984 vs 810 genes) we consider the enrichment results largely similar. At the 0.05% tail for influenza we expect 4.568 genes and observe 14 (p-value = 0.00014, FDR = 0.00112), whereas for HIV/SIV we expect 4.323 genes and observe 11 (p-value = 0.00398, FDR = 0.01854). Of note, these two categories overlap substantially, with 36% of the 0.05% tail candidate genes in the influenza VIP set being also HIV/SIV VIPs, which makes it difficult to establish the independence of their signatures. Not surprisingly other VIP categories also show some evidence of positive selection. Ebola is an interesting example, although it is a very small category with substantial overlap with HIV/SIV: three of the four 0.05% tail candidate genes in the ebola VIPs set are also HIV/SIV VIPs. Thus, evidence for other VIP categories exist, but together our results point to HIV/SIV as a particularly important selective force in chimpanzees.

Finally, we explore other biological categories in a hypothesis-free analysis. An enrichment test of GO categories [48] reveals 25 significantly enriched GO categories, two of which are relevant to host-viral interactions: ‘IκB/NFκB complex’ (0.5% candidates threshold, p-value = 0.00008) and ‘positive regulation by host of viral transcription’ (0.5% candidates threshold, p-value = 0.00002) (see rest of categories and FDR values in Fig D in S1 Appendix). Notably, as discussed below, these two categories are intimately involved in the host biology under SIV/HIV infection. As expected, this analysis indicates likely adaptations to selective forces beyond SIV, although no category is as consistently enriched across thresholds as the HIV/SIV VIPs or SIV-response categories.

Thus, the potential targets of positive selection in the central-eastern ancestral population show enrichment patterns expected under adaptation to SIV/HIV and point to physical protein interactions (VIPs) and initial immune reaction (SIV-response) as likely being key in early adaptations to zoonotic SIV. Interestingly, the candidate windows are not enriched in existing HIV-related GWAS hits when enrichment is investigated with Gowinda as above. This suggests that while association studies of AIDS-related traits can identify genetic variants involved in clinical phenotypes (often with treatment), they do not reveal the genetic bases of the biological mechanisms that may allow natural hosts to control the pathogenicity of the virus.

### Potential sweeps of non-synonymous variants in *CD4*

**Fig 4:**
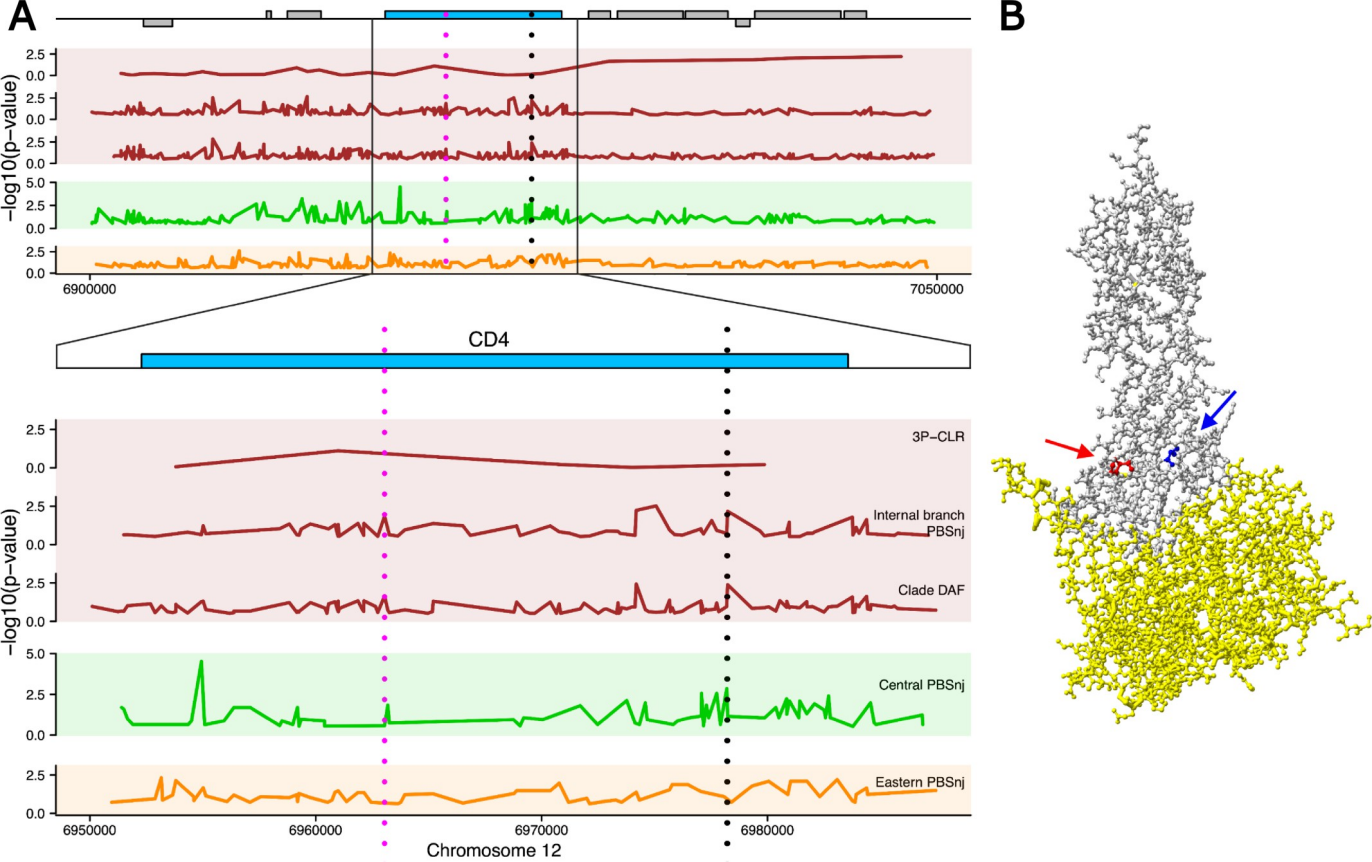
Signatures of selective sweeps in the extended *CD4* locus. **A.** Genomic representation of 150 kb and a magnified view of *CD4* with signatures of positive selection from 3P-CLR, PBSnj and clade-DAF. Statistics are coloured by population. The vertical dotted lines indicate best candidate SNPs of interest. The pink line indicates a splice site synonymous variant at position 6963043 with signatures of positive selection in the central-eastern ancestor. The black dotted line indicates two SNPs 39bp apart: a missense SNP in position 6978193 (the V55I SNP) with signatures of positive selection in centrals, and a missense SNP at position 6978232 (the P68T SNP) with signatures of selection in the central-eastern ancestor. For the DAF of these candidate SNPs across chimpanzee subspecies see Fig E in S1 Appendix. **B.** Protein structure diagram showing the first two CD4 domains (grey) bound to the HIV envelope protein (yellow) [50] and the amino acids corresponding to the two missense SNPs of interest, 6978193 (V55I) in blue and 6978232 (P68T) in red.

A fundamental gene in SIV pathology is *CD4*, which encodes the glycoprotein required for HIV/SIV cell entry, additional to a chemokine coreceptor [38]. Excitingly, *CD4* lies within a candidate window at the 0.5% 3P-CLR threshold, showing signatures of positive selection in the central-eastern ancestral population. *CD4*’s protein-coding genomic region also contains SNPs with high derived allele frequencies in the central-eastern clade that are also highly differentiated compared with Nigeria-Cameroon (Fig 4A), as well as multiple signals of positive selection both in central and eastern chimpanzees (with PBSnj, data from Schmidt *et al*., [32]) (Fig 4A). These signatures provide support for positive selection driving the evolutionary history of the gene in chimpanzees.

Bibollet-Ruche *et al*., [49] have shown that CD4 harbours functionally relevant variants in chimpanzees, so to integrate both types of information and better understand the gene’s signatures of selection we aimed to localise the most likely selected variant(s). We focused on SNPs with the highest allele frequency differentiation between the two chimpanzee clades (central-eastern and Nigeria-Cameroon-western), as they are prime candidates to explain the selective sweeps in the central-eastern ancestor identified by 3P-CLR (see Methods). We identified these SNPs with PBSnj in the internal branch (representing the central-eastern ancestor) with data from [32] and combine this with information on the amino acids found to determine SIV infectivity of chimpanzee cells by Bibollet-Ruche *et al*., [49]. The P68T variant (chr12:6978232) falls in the tail of the empirical PBSnj distribution (PBSnj p-value = 0.0066), and is among the SNPs with the highest PBSnj value in this window. It is one of only four CD4 amino acid polymorphisms known to determine SIV infectivity of chimpanzee cells [49] being located, in the folded protein, near the binding site between CD4 and SIV’s *env* protein (Fig 4B). Central and eastern chimpanzees carry the ancestral P allele, able to inhibit SIVcpz. The P68T variant is a prime candidate to drive the selective sweep we observe in the central-eastern ancestor, with selection driving the P allele, though it remains possible that selection acted on a different genetic variant, or on multiple.

Interestingly, P68T is not the only *CD4* functional variant with signatures of selection in chimpanzees. The V55I variant (chr12:6978193) (Fig 4A) is not strongly differentiated in this branch (PBSnj p-value = 0.1217), but has PBSnj signatures of positive selection in central chimpanzees (central PBSnj p-value = 0.0014). Like P68T, in the folded protein V55I is near the binding site between CD4 and SIV’s *env* protein (Fig 4B), and it is likely also functionally relevant, though the evidence from Bibollet-Ruche *et al*., [49] is weaker than for P68T. We note that in addition, a splice site region synonymous variant (chr12:6963043) is also among the most highly differentiated SNPs in the internal branch according to PBSnj (Fig 4B). It seems clear that CD4 being a key player in SIV infection, has been targeted by positive selection recurrently and in complex ways. Together with the three chemokine co-receptors showing signatures of positive selection in central chimpanzees [32], this indicates that proteins involved in SIV cell entry have evolved under adaptive evolution across chimpanzee populations.

### SIV-mediated selection across populations

Our results thus indicate that SIV was a strong selective force in the common ancestor of central and eastern chimpanzees. This adds to previous evidence of SIV-related adaptation both in the central and eastern populations [32]. To understand how chimpanzees have been able to adapt to SIV thus requires integrating information across these populations. We integrated selection scores across the three populations (3P-CLR for the central-eastern ancestral population and previously computed PBSnj for the central and eastern subspecies [32], Fig 1) and tested as above if any SIV-related biological categories have been targeted by positive selection across populations using Gowinda (Fig F in S1 Appendix).

The VIPs HIV/SIV category shows nominal enrichment across all possible configurations of populations: the central-eastern ancestor and central subspecies (p-value = 0.00894, BH-FDR = 0.185535), the central-eastern ancestor and eastern subspecies (p-value = 0.00662, BH-FDR = 0.1855350), and the three populations together (central-eastern ancestor, central and eastern subspecies; p-value = 0.0161, BH-FDR = 0.2700533). None of these are significant after correcting FDR values for the number of populations tested using a Benjamini and Hochberg (BH) correction; however, Gowinda is underpowered in this scenario, as several genes show signatures of positive selection in multiple lineages, but are counted only once. To address this limitation, we developed a custom method, ‘*set_perm*’, which extends the functionality of Gowinda to test if the joint distribution of candidate loci from different populations reveals enrichment for particular biological functions (Methods, S2 Appendix). Focusing on the three populations together, *set_perm* reveals significant enrichment (after correcting FDR values for testing multiple populations using a BH correction) for the VIPs HIV/SIV category (105.37901 expected, 145 observed, p-value = 0.00003, BH FDR = 0.00159), SIV-response genes (276.44037 expected, 308 observed, p-value = 0.01678, BH FDR = 0.04604) and the green SIV co-expression module (8.4254 expected, 20 observed, p-value = 0.00039, BH FDR = 0.02241) (Fig G in S1 Appendix). This green co-expression module is one of the two showing evidence of positive selection in the central-eastern ancestor, and as mentioned above is associated with an acute response to SIV [26]. The higher power of *set_perm* when genes have signatures in several populations means that additional categories also become significant, including VIPs for influenza (112.18832 expected, 145 observed, p-value = 0.00061, BH FDR = 0.01815) and VIPs for dengue viruses (9.40297 expected, 20 observed, p-value = 0.00083, BH FDR = 0.02742).

**Fig 5:**
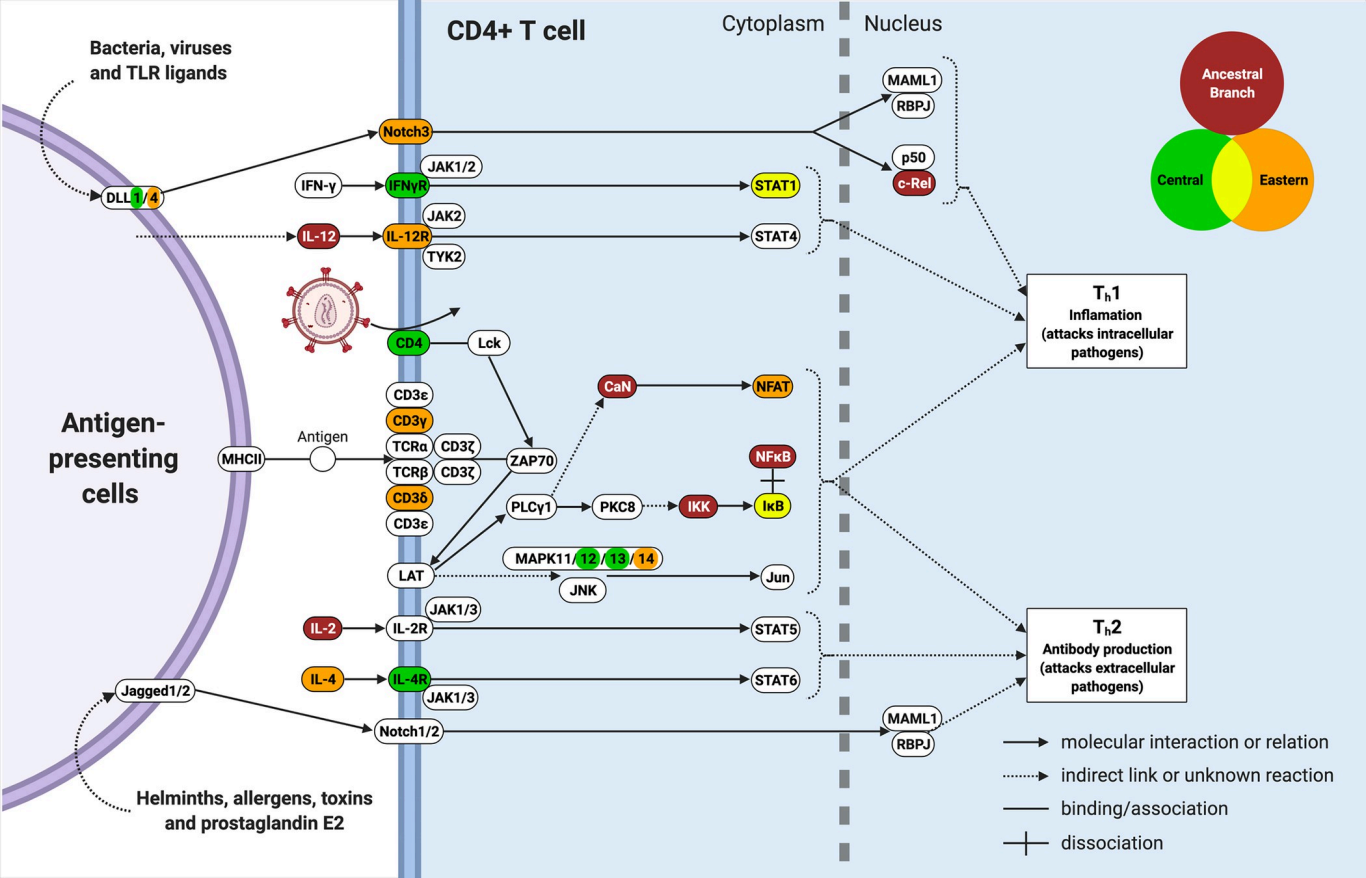
Simplified diagram of the Th1/Th2 cell differentiation pathway, highlighting proteins encoded by genes with signatures of positive selection. Proteins are coloured according to the population(s) in which they show signatures of positive selection (see legend), with white indicating proteins with no signatures of positive selection. Proteins in contact with other proteins indicate protein complexes. *IL13* is a candidate in both central and eastern but is not included in this simplified diagram. *RELA* is a candidate gene in the central-eastern ancestor and codes for a protein subunit of both NFκB and c-Rel. This diagram was created with BioRender.com.

To explore more generally which biological processes have been targeted by positive selection, we also tested for enrichment of signatures of positive selection in KEGG pathways [51]. The KEGG pathway database consists of manually drawn molecular interaction diagrams for a wide range of biological pathways [51]. Using Gowinda, only one pathway exhibits nominal enrichment in the combined candidates from the three populations (central-eastern ancestor, central and eastern subspecies): “Th1/Th2 cell differentiation pathway” (10.6 expected, 22 observed, p-value = 0.00056, BH-FDR = 0.25552) (Fig H in S1 Appendix and Table A in S1 Appendix). Underpinning this signal are 22 genes: five with signatures of positive selection in the central-eastern ancestor, eleven in eastern and nine in central chimpanzees, of which three genes are candidates both in eastern and central chimpanzees (Fig 5). This result is confirmed and strengthened using *set_perm*, where this pathway shows a strongly significant enrichment even after correcting for testing multiple populations: (“Th1/Th2 cell differentiation pathway”, 10.2 expected, 25 observed, p-value = 0.00002, BH-FDR = 0.013800) (Fig I in S1 Appendix). The similar contribution of signatures of selection in the three populations shows that the Th1/Th2 pathway has likely repeatedly undergone selection through time, although the individual genes targeted have largely differed across the three populations. This pathway is intimately associated with SIV infection, being active only in CD4+ T cells, which are the only cells that SIV infects (since CD4 is required for cell entry). Moreover, SIV coordinates its replication with T cell activation, as differentiation of CD4+ T helper cells involves upregulation of the transcription factors required to transcribe HIV/SIV genes [52,53]. By this process, HIV replication leads to pathogenesis via the destruction of CD4+ T cells and the development of AIDS [54]. Finally, the Th1/Th2 cell differentiation pathway is critical to generate an efficient immune response against viruses such as HIV/SIV (see Discussion). Of note, multiple genes with signatures of positive selection in this pathway encode for proteins that play critical roles in SIV/HIV infection (Fig 5). These include the transcription factors Nuclear Factor Kappa B (NFκB) and Nuclear Factor of Activated T-cells (NFAT) (see Discussion).

The higher power of *set_perm* means that it reveals additional significantly enriched KEGG categories (Fig I in S1 Appendix). For example, in the three populations together the immune-related ‘primary immunodeficiency’ category becomes significant (2.0645 expected, 10 observed, p-value = 0.00003, BH FDR = 0.006900), although it has only eight significant genes, half of them in centrals. Interestingly ‘Epstein-Barr virus infection’ (18.09451 expected, 37 observed, p-value = 0.00003, BH FDR = 0.013800) and ‘leishmaniasis’ (5.61591 expected, 17 observed, p-value = 0.00006, BH FDR = 0.016848) pathways also show significant enrichment in the combined three populations. These are not biological networks but rather pathogen-associated categories that might possibly point to additional selective pressures. Still, we note that 69.2% of the significant ‘leishmaniasis’ genes belong also to the larger Th1/Th2 cell differentiation pathway. Thus, to what extent this signature is fully independent from those in the Th1/Th2 cell differentiation pathway is unclear.

In summary, our genome-wide analysis shows strong evidence of positive selection in the common ancestor of central and eastern chimpanzees for SIV-response genes, SIV co-expression modules, and HIV/SIV VIPs (together with VIPs for other viruses). Further, when combining candidates from the central-eastern ancestor with those of its daughter subspecies, we find evidence of positive selection in each of these SIV-related categories, together with recurrent positive selection in immune-related pathways, notably the “Th1/Th2 cell differentiation pathway”.

## Discussion

SIV is a formidable selective force, which has seemingly driven genetic adaptation in chimpanzees since zoonotic infection until recent times. Here we identify adaptations to SIV in the central-eastern ancestor. Together with our previous work showing evidence of positive selection in multiple SIV-related genes in central and eastern chimpanzees, but not in western and Nigeria-Cameroon [32], this supports the notion that zoonotic transmission took place in the central-eastern ancestor and that SIV immediately became a strong and continuous selective pressure in infected populations.

In the central-eastern ancestral population, loci with the strongest evidence of positive selection in the genome are significantly enriched in two sets of genes that are intimately associated with SIV, although in substantially different ways: genes that change expression upon SIV infection (‘SIV-response genes’ and ‘SIV co-expression modules’) and genes that encode proteins which physically interact with HIV/SIV (‘VIPs’). We also find evidence of positive selection driving sequence evolution of CD4, a protein with a critical role in immunity which is necessary for SIV cell entry. This reveals how diverse the initial adaptation to SIV likely was, consisting of genetic changes in host proteins involved in the host response, physical interaction and cell entry of SIV.

Notably, only the VIPs show an enrichment in signatures of positive selection only in the ancestral population, with the other categories showing evidence of selection at later times too. It is easy to imagine why VIPs may be under strong selective pressure after first infection, as they physically interact with the virus, while other mechanisms of adaptation may become more relevant at later times.

Genes involved in the host response to SIV in natural hosts (SIV-response genes) show evidence of positive selection not only in the central-eastern ancestral population, but also in eastern chimpanzees [32]. Combined, these observations suggest that the modulation of the host response through gene expression evolves immediately after zoonotic transmission, and continues over time. This idea agrees well with the fact that SIV-response genes evolve under positive selection also in vervet monkeys [26], which are long-term SIV natural hosts. It is interesting that SIV-response genes were not identified experimentally in chimpanzees, but in vervet monkeys, [26,39,40]. Their apparent role in adaptation in both species is consistent with the idea that host tolerance to SIV may evolve via similar mechanisms across distantly related species.

Genes that encode for the few proteins required by SIV to enter the cell also show evidence of positive selection across populations. Bibollet-Ruche *et al*., [49,55] showed that CD4, which is the required primary receptor for SIV cell entry, harbours multiple functionally relevant genetic variants. Complementing this important work, we find evidence of positive selection in the central-eastern ancestor at *CD4* with the most likely selected variant, P68T, which we hypothesise underwent a soft sweep in the central-eastern ancestor, being among only four CD4 missense variants shown to modify SIV infectivity in chimpanzee cells [49]. Of note, with both alleles chimpanzee P68T inhibits SIVcpz compared with the human CD4, though the derived T allele is most inhibitory [49]. The T allele is fixed in western chimpanzees while Nigeria-Cameroon chimpanzees are polymorphic (0.55) and bonobos and humans harbour the P allele, which is ancestral [49,55]. It is thus likely that the site was polymorphic in the common ancestor of all chimpanzees, making selection on P68 in the central-eastern ancestor likely to have occurred on standing variation–although it remains possible that selection on a different variant is responsible for the signatures of selection in CD4. It is nevertheless interesting that the site is invariant in western chimpanzees. Bibollet-Ruche *et al*., [49] proposed that fixation of the T allele in western chimpanzees could be due to adaptation to an SIV-related retrovirus. This is an interesting hypothesis that we are able to test using a formal test of positive selection in western chimpanzees, the PBSnj statistic in the western branch from Schmidt *et al*., [32]. Under our conservative 0.5% critical value western chimpanzees show no significant evidence of positive selection at P68T (PBSnj western branch p-value = 0.0218). Still, this SNP falls within the 5% tail of the empirical distribution, and under less stringent criteria would be considered to harbour signatures of positive selection. Thus, while we cannot discard the possibility that the lack of diversity at P68T in western chimpanzees is explained by random genetic drift, which is strong in this subspecies due to their small effective population size and bottlenecks [43], our results suggest that the fixation of the T allele in western chimpanzees could be due to positive selection. CD4 has a critical role in immunity and has been suggested to have some signatures of positive selection in species without historical infection by SIV (e.g. humans [56]). Given the absence of natural SIV infections in western chimpanzees it appears more likely that a different virus, perhaps a related retrovirus, may have driven selection in this subspecies. In any case, we note that the allele frequency change in P68T in western chimpanzees has no effect over the signatures of positive selection in *CD4* in the central-eastern ancestor (we used Nigeria-Cameroon as the outgroup).

Beyond P68T, *CD4* harbours signatures of positive selection in other SNPs and subspecies. As mentioned in the Results, the V55I variant, which due to its position in the protein is likely to have functional effects, has PBSnj signatures of positive selection in central chimpanzees. Further, a C to T SNP within *CD4* (chr12:6973383) has significant signatures of positive selection in Nigeria-Cameroon (PBSnj p-value = 0.002) (data from [32]). This adds to a picture of multiple sweeps, both ancestral and lineage-specific, in *CD4*. Although outside of the scope of this paper, an extensive analysis of this complex region that integrates functional and evolutionary information would be extremely interesting. Remarkably, the multiple signatures of positive selection in *CD4* add to existing evidence of adaptation in central chimpanzees in *CCR3*, *CCR9* and *CXCR6* [32], which mediate SIV/HIV cell entry [33–35] together with the primary receptor CD4.

Together, our results reveal that combining information across time and populations is critical to understand chimpanzee adaptation to SIV. By integrating selection scores from the central-eastern ancestor with those from the central and eastern subspecies, we identify strong enrichment in the Th1/Th2 cell differentiation pathway. Twenty-two genes in the Th1/Th2 cell differentiation pathway have signatures of positive selection across time, three genes in two separate populations. Together these observations show that this molecular pathway has been repeatedly hit by positive selection over time. Why this pathway? First, the Th1/Th2 cell differentiation pathway is critical for immunity against intracellular pathogens, including viruses. Naïve CD4+ T cells recognise a MHC class II peptide, are activated and divide to give rise to clone effector CD4+ T cells specific for that antigen [57]. CD4+ T cells can differentiate into T helper type-1 (Th1), T helper type-2 (Th2), or other T helper types, each with distinct cytokine-secretion phenotypes, production of distinct interferons, and different downstream immune responses [57]. By shaping which type of helper cell a CD4+ T cell will differentiate into, this pathway has critical effects on immunity.

Second, the Th1/Th2 cell differentiation pathway is particularly relevant for HIV/SIV pathogenesis, especially for control of viral replication. This is illustrated by the fact that a unique subset of humans, ‘HIV controllers’, who are able to spontaneously control HIV infection without treatment, are characterised by a Th1 differentiation bias [58,59]. Specifically, HIV controllers differentiate more naïve CD4+ T cells into Th1 than into other T helper subsets [58,59]. *In vitro* studies demonstrate that Th1 cells are more resistant to HIV replication than Th2 cells, since their higher expression of *APOBEC3G* limits reverse transcription and integration of HIV virions in Th1 better than in Th2 cells [60,61]. It thus seems natural that biasing the Th1/Th2 cell differentiation pathway towards Th1 differentiation may be an efficient route to control SIV/HIV pathogenicity.

Identified candidate targets of selection include key transcription factors required for SIV/HIV replication: NFκB (in central-eastern ancestor) and NFAT (in easterns). NFAT proteins and HIV-1 upregulate each other and may allow establishment of HIV-1 in the early stages of infection [62]. Both NFκB and NFAT bind to the same site which is identical in HIV-1 and HIV-2. We find signatures of positive selection in genes encoding proteins which inhibit (IκB in centrals and easterns) and activate (IKK in central-eastern ancestor) the NFκB protein complex. SIV/HIV have evolved to manipulate NFκB and IκB to minimise antiviral gene expression, while allowing NFκB-induced viral transcription [63–65]. Hence, genes encoding NFκB, IκB and their immediate interactors are plausible targets for selection [63–65].

Our results contribute to the growing literature indicating that SIV has been and continues to be a strong selection pressure in chimpanzee evolution. Previous studies have largely focused on possible SIV-mediated selection by characterising variation at the MHC. Evidence of an ancient selective sweep at MHC-I in the ancestor of chimpanzees and bonobos was initially suggested to have been driven by SIV [27–29] but is now attributed to an ‘SIV-like retrovirus’, given such an ancient SIVcpz origin (and subsequent loss of the virus in three lineages) is very unlikely [12,66]. Whereas, evidence that SIV drives adaptation at MHC-I in the very recent past has been found by monitoring allele frequencies through time in eastern communities with high and low SIV loads [67]. We do not find evidence of positive selection at MHC-I in the central-eastern ancestor, although we likely have low power to identify signatures of selection in this highly complex region. Further, it is likely that balancing selection is acting on the MHC, as diversity at these sites can be protective against SIV and other viruses [68], further complicating the picture. We note that a key novelty of our study is that the genomic dataset allowed us to go beyond the few genes known to play important roles in SIV/HIV infection and perform formal tests for selection genome-wide. Our approach provides compelling evidence of SIV-mediated selection only in the central-eastern chimpanzee clade.

Although we have focused on selection in response to SIV, additional selective pressures were surely relevant for the fitness of the central-eastern ancestor, and later populations. Here, in the central-eastern ancestor, we also identify significant enrichments of signatures of selection in VIPs that interact with influenza and other viruses, including ebolaviruses (Fig 3). Ebolaviruses have been implicated in disease and mortality of wild chimpanzees [69]. Influenza can infect captive chimpanzees [70–72] and although respiratory and ‘flu-like’ diseases have been detected in wild chimpanzees, we are not aware of any study that has specifically detected influenza in such cases [73–75] and those would in any case represent contemporary infections. Nevertheless, it is possible that an archaic influenza-like virus infected chimpanzees thousands of years ago, leaving its mark in the genome in the same way as an unknown archaic SARS-CoV-2-like virus has been proposed to leave a signature of genetic adaptation in Asian human populations [76]. In the future it would be interesting to explore the biological consequences of other targets of selection identified in our candidate windows.

Still, we find overwhelming evidence that SIV has been a strong selective force in central and eastern chimpanzees and their common ancestor, consistent with the scenario of zoonotic transmission of SIV into this ancestral population. This includes initial adaptations in SIV-interacting proteins, combined with adaptations on SIV response genes and cell entry genes that continued into daughter populations, and with adaptations in the Th1/Th2 cell differentiation pathway over time. This suggests the development of natural host immunity to SIV likely requires adaptation in the host-virus interacting factors and the factors that mediate cell entry and the immune response to infection. All of this strongly implies that SIV mediated selection has and continues to be important in the evolution of central and eastern chimpanzees, and that we can identify these adaptations with population genetics.

## Methods

### Simulations and power analysis

For 3P-CLR power analysis, we performed forward-in-time simulations in SLiM v.3.2 [77,78]. Following the inferred *Pan* demographic model [43,79] we simulated regions of length 1.2Mb, under neutrality and with positive selection. We assumed a 25-year generation time [80], 0.96x10^-8^ recombination rate [43,79] and a Wright-Fisher model. We performed 1,000 neutral simulations and 1,000 simulations for both selection coefficients (*s* = 0.1, 0.05). Our selection simulations followed a hard sweep, conditional on fixation before the central-eastern subspecies split (106 kya). Therefore, we sampled only a subset of all possible evolutionary trajectories of the beneficial mutation. Following Racimo [41] every 10^th^ SNP was a focal SNP, around which a 0.25cM window was centred, sliding every 10 SNPs. 100 SNPs were randomly sampled per window and used to calculate the 3P-CLR statistic, to test each window for selection signatures. For computational efficiency we extended 3P-CLR to allow the method to analyse 5kb segments of each chromosome. Results for the original and extended source codes show the expected high correlation (Fig J in S1 Appendix). For the distribution of 3P-CLR scores for simulated under neutrality and positive selection see Fig K in S1 Appendix. Nigeria-Cameroon was used as an outgroup to the central and eastern target populations. We used Nigeria-Cameroon rather than western who exhibit higher levels of drift and lower levels of segregating polymorphism [43]. Hence using western as the outgroup would reduce the number of sites we could investigate, as 3P-CLR only considers sites which are segregating in the outgroup [41]. We also note that gene flow between Nigeria-Cameroon and central-eastern chimpanzees may mask some signatures of selection, but would not generate them. We produced ROC curves to visualise 3P-CLR’s sensitivity and specificity, using pROC [81] in R v.3.5.2 [R Core Team 2018].

### Identifying selection in the central-eastern ancestor

We analysed genomic data generated by De Manuel et al. [43], using the same 3P-CLR parameters from our power analysis. This data consists of 47 genomes sequenced to high coverage (mean 22.5-fold coverage per individual), which had been sampled from chimpanzees of known subspecies: 18 central, 19 eastern, 10 Nigeria-Cameroon chimpanzees. We used the EPO alignment to infer the ancestral allele for calculating derived allele frequencies. We used the *Pan* diversity recombination map [44] to obtain genetic distances from physical distances. For the empirical distribution of 3P-CLR scores genome-wide and in the tails of the distribution see Fig L-M in S1 Appendix.

### *Post-hoc* SFS analysis of candidate windows in the central-eastern ancestor

We used the VCF file from De Manuel et al. [43] to calculate the unfolded SFS for the whole genome and for each 3P-CLR candidate window threshold. Sites were included only if they were polymorphic when considering central, eastern and Nigeria-Cameroon i.e. western specific variants were excluded. Combined central and eastern allele frequencies were calculated by simply pooling the samples as each subspecies had almost identical sample sizes (central: 18, eastern: 19). The difference in allele frequencies between central-eastern and Nigeria-Cameroon was calculated as the absolute difference in derived allele frequencies (DAF) between central-eastern and Nigeria-Cameroon for each site. The minimum DAF at which we can identify a polymorphism depends on the sample size. We have 18 central and 19 eastern samples, and so the minimum DAFs possible are as follows—central: 0.028, eastern: 0.026, central-eastern combined: 0.014.

### Gene set enrichment analyses in the central-eastern ancestor

We tested our 3P-CLR candidate windows for gene set enrichments, using Gowinda v.1.12 [45], at the 3 highest quantiles of likelihood scores (0.5%, 0.1% and 0.05% candidate windows), as power to detect significant gene enrichment varies with the number of genes sampled. We tested for enrichment in different gene sets: SIV-response genes, SIV co-expression modules, VIPs, GO categories and KEGG biological pathways [26,39,40,46–48,51]. We note that the SIV co-expression modules are named as colours, consistent with [26]. Gowinda provides a p-value for the likelihood of finding the same evidence of selection seen in the candidate genes in a random set of genes, when considering genes of equivalent length and localisation in the genome [45]. These sets of random genes provide the appropriate negative controls for the analysis. Gowinda also calculates an FDR for each p-value. All genes tested were 1–1 human homologs and the analysis was run in ‘gene’ mode.

### Overlap with all PBSnj SNPs in the internal branch

To identify the SNPs with the strongest evidence of selection, we selected those with the largest allele frequency difference between the central-eastern and the western-Nigeria-Cameroon clades, within each significant 3P-CLR window at the 0.5% threshold. PBSnj [32] was used to assess allele frequency difference between the two clades. We then filtered by the 20 SNPs with the highest PBSnj value per window. This results in 10,676,926 SNPs genome-wide, which we annotated using ENSEMBL for pantro2.1.4, variant effect predictor [82] and regulomeDB [83]. Functional annotation of the 20 highest PBSnj SNPs per window at the 0.5% threshold are shown in Fig N-O in S1 Appendix. SNPs of interest, additional to those identified in *CD4*, are shown in Table B in S1 Appendix. We also calculated the difference in derived allele frequencies between the two clades (clade-DAF), as the difference in mean-weighted derived allele frequencies. The positions of amino acids within the structure of CD4 coded for by SNPs of interest were plotted using iCn3D v3.1.1 structure viewer [84].

### Identifying repeated targets of selection across branches

To investigate the targets of selection across time and populations, we combined selection candidates identified in the central-eastern ancestor with those previously identified in the central and the eastern subspecies by [32]. To ensure each population is equally represented in the analysis, we assigned a single empirical p-value to each gene for each population and used genes in the 0.5% threshold for the central-eastern ancestor, to have an equal number of candidate genes as for the subspecies. Total number of candidate genes per population is as follows: in the central-eastern ancestor (817 genes), central p < 0.000192 (816 genes), eastern p < 0.000228 (806 genes). We tested for enrichment of our combined candidate sets in the same gene sets as above: SIV-response genes, SIV co-expression modules, VIPs, GO categories and KEGG biological pathways [26,39,40,46–48,51] using Gowinda [45]. However, Gowinda is underpowered when testing for enrichment in multiple lineages.

In order to perform gene set enrichment tests on more than one lineage, we used a new method developed by JMS. ***set_perm*** implements a permutation-based enrichment test that for a single lineage is equivalent to Gowinda [45] but that correctly accounts for genes showing signatures of natural selection in more than one lineage. Details of the method can be found in the S2 Appendix and the code is available at https://github.com/joshuamschmidt/set_perm).

Briefly, a joint test of lineages is performed by summing the number of candidate genes per gene set across lineages. Thus, the total observed number of genes is simply the sum of the observed number of genes across the different lineages, per gene set. Note, that this means that the same gene can be counted more than once, if it is a candidate in more than one lineage.

Permutations are performed by first generating independent random gene sets for each lineage, and then combining them into a joint set across all lineages. Thus, in this case, for each gene set (S) and permutation (i), the joint permutation set, *joint_Si_*, is obtained by summing the number of genes (n) per lineage (k) i.e.


$$joint_{Si}=\sum_{1}^{k}n_{k}$$

The p-values and FDR-corrected significance are calculated from permutation sets as described for Gowinda [45]. As above, any gene present in more than one independent permutation test will contribute more than once to the joint permutation test. Note, the number of observed genes may differ slightly between Gowinda and *set_perm*. This is because, in order to perform joint lineage testing with Gowinda we define candidates at the gene level, whereas with *set_perm* candidates are defined at the SNP level.

## Supporting information

S1 Appendix **Supplementary Figures A-O and Tables A,B. Fig A in S1 Appendix. Power of 3P-CLR in the ancestral central-eastern population**. Each ROC curve was generated from 1000 neutral simulations and 1000 selection simulations for each s (s = 0.05, 0.1). **Fig B in S1 Appendix. Full unfolded SFS for the whole genome and each 3P-CLR tail threshold in centrals (top left), easterns (top right) and central-eastern combined (bottom).** The central-eastern combined SFS was made by simply pooling all the samples as both subspecies have nearly identical sample sizes (central: 18, eastern: 19). The SFS are all indicative of selective sweeps. **Fig C in S1 Appendix**. Site frequency spectrum of SNPs in candidate windows. Allele frequencies of SNPs genome-wide and at different 3P-CLR tail thresholds. A: Unfolded SFS for central, eastern and central and eastern combined. The X axis is limited to focus on high-frequency derived alleles B: Absolute DAF difference between central-eastern and Nigeria-Cameroon. **Fig D in S1 Appendix. Enrichment of gene ontology (GO) categories across candidate genes at different 3P-CLR quantiles in the central-eastern ancestor**. Only categories with a significant enrichment in at least one quantile (FDR<0.05) are shown. Categories are separated by GO class: Biological Process, Cellular Component and Molecular Function. Colours represent FDR values (red as highest significance). Grey represents instances where a GO category was undetected in that particular quantile. Stars indicate a significant enrichment in that 3P-CLR quantile. **Fig E in S1 Appendix. DAF of the three candidate SNPs of interest in *CD4* across chimpanzee subspecies.** These candidate SNPs correspond to those highlighted in Fig 4. SNP at chr12:6963043 represents a splice variant with signatures of positive selection in the central-eastern ancestor. SNPs at chr12:6978193 (V55I SNP) and chr12:6978232 (P68T SNP) are missense variants with signatures of positive selection in centrals and the central-eastern ancestor respectively. **Fig F in S1 Appendix. Enrichment in SIV-related, VIP and GO categories of candidate targets of positive selection across populations, tested using Gowinda**. Abbreviations indicate the populations tested: central-eastern ancestor + central (A+C), central-eastern ancestor + eastern (A+E), central-eastern ancestor + central + eastern (A+C+E). Categories are separated by gene set tested: SIV responsive genes, SIV co-expression modules, VIPs and GO categories [26,39,40,46–48]. Colours represent Benjamini and Hochberg corrected-FDR values (red as highest significance). We note that no category reaches significant enrichment (after correcting FDR values for the number of populations tested using a BH correction). **Fig G in S1 Appendix. Enrichment in SIV-related, and VIP categories of candidate targets of positive selection across populations, tested using *set_perm*.** Abbreviations indicate the populations tested: central (C), eastern (E), central-eastern ancestor (A), central-eastern ancestor + central (A+C), central-eastern ancestor + eastern (A+E), central-eastern ancestor + central + eastern (A+C+E). The following categories are significantly enriched in the three populations together at BH-corrected FDR < 0.05: SIV responsive genes, SIV co-expression green module, HIV/SIV VIPs, influenza (IAV) VIPs and dengue (DENV) VIPs.All of these categories are also significantly enriched when combining the central-eastern ancestor and the eastern subspecies. The SIV co-expression green module is significantly enriched when combining the central-eastern ancestor and the central subspecies. In the single lineages, we see significant enrichment in the eastern subspecies in the SIV-responsive and HIV/SIV VIPs. While the central-eastern ancestor is significantly enriched in the SIV co-expression green module, HIV/SIV VIPs and influenza (IAV) VIPs. **Fig H in S1 Appendix. Enrichment in KEGG pathways of candidate targets of positive selection across populations, tested using Gowinda**. Abbreviations indicate the population(s) tested: central-eastern ancestor (A), central-eastern ancestor + central + eastern (A+C+E). For the central-eastern ancestor we used candidate genes in the least stringent quantile (0.5), to match the number of candidates for the subspecies. Colours represent BH-corrected FDR values (red as highest significance). We note that none of the KEGG pathways reaches BH-corrected FDR < 0.05. The strongest enrichment is for the Th1 and Th2 cell differentiation pathway for A+C+E with p-value = 0.00056 and BH-FDR = 0.25552, which represents a nominal enrichment. **Fig I in S1 Appendix. Enrichment in KEGG pathways of candidate targets of positive selection across populations, tested using *set_perm*.** Abbreviations indicate the populations tested: central-eastern ancestor + central (A+C), central-eastern ancestor + eastern (A+E), central-eastern ancestor + central + eastern (A+C+E). The following KEGG categories are significantly enriched in the three populations together at BH-corrected FDR < 0.05: ’Th1 and Th2 cell differentiation’, ’primary immunodeficiency’, ’Epstein-Barr virus infection’ and ’leishmaniasis’. The ’primary immunodeficiency’ and ’leishmaniasis’ categories are also significantly enriched in the central subspecies. Hence for the ’primary immunodeficiency’ and ’leishmaniasis’ gene sets, the central lineage is likely driving the signal when the three populations are combined. When the central-eastern ancestor and eastern subspecies are combined, we see significant enrichment in the ‘chromosome and associated proteins’ pathway, which is also significantly enriched in the central-eastern ancestor. **Fig J in S1 Appendix. Correlation between the 3P-CLR values with the original and extended code.** Likelihood scores are significantly correlated between the original and modified 3P-CLR source code (rho = 0.4565, p<2.2e-16). The absence of a perfect correlation is not due to differences in the algorithm, but due to the sampling variance of SNPs in each window. Specifically, if more than 100 SNPs are present within a given window, 3P-CLR chooses 100 SNPs at random. Thus, the same SNPs will not be sampled each time the method is run. Variation in 3P-CLR likelihood ratio scores therefore results if different SNPs were used to calculate the statistic. As expected, the correlation is weaker for low 3P-CLR but high at higher 3P-CLR values, where candidates of positive selection fall. **Fig K in S1 Appendix. Distribution of 3P-CLR scores** for data simulated under neutrality (A) and positive selection with selection coefficients of 0.05 (B) and 0.1 (C), 1000 replicates were generated in each case. **Fig L in S1 Appendix. Empirical distribution of 3P-CLR scores**. Vertical lines indicate the thresholds used to define candidate windows (0.5%, 0.1% and 0.05% tails of the empirical distribution). **Fig M in S1 Appendix: Distribution of 3P-CLR scores in the tails of the empirical distribution.** (A) the 0.5%, (B) 0.1% and (C) 0.05% candidate quantiles, corresponding to 4090, 818 and 409 genomic windows respectively. **Fig N in S1 Appendix: Functional annotation of 20 highest PBSnj SNPs per window at the 0.5% threshold using VEP**. **Fig O in S1 Appendix: Functional annotation of 20 highest PBSnj SNPs per window at the 0.5% threshold using regulomeDB**. **Table A in S1 Appendix. Number of candidate genes from each population which belong to gene categories of interest.** The number of genes which are candidates in central only, eastern only, and ancestral only are shown in the C, E and A columns respectively. The number of genes which are candidates in both central and eastern, central and ancestral, eastern and ancestral, or all three populations are shown in the C+E, C+A, E+A, and C+E+A columns respectively. **Table B in S1 Appendix. SNPs of interest in genes identified within candidate windows of positive selection at the 0.5% threshold in the central-eastern ancestor, additional to *CD4*.** SNPs with regulomeDB scores of 1f, 2a, 2b, 2c are considered to have putative significant regulatory function, due to the lack of eQTL data for chimpanzees. Category indicates the gene set each gene belongs to. Abbreviations: HCMV and ADV indicate human cytomegalovirus and adenovirus respectively.
(DOCX)

S2 Appendix Supplementary note of *set_perm* method. (DOCX)

S1 Enrichment Tables Supplementary tables of gene set enrichment results. (XLSX)
