# Supplementary material for: Genetic adaptations to SIV across chimpanzee populations
Source: PLoS Genet. 2022 Aug 25;18(8):e1010337. doi: 10.1371/journal.pgen.1010337 (PMC9467346; doi:10.1371/journal.pgen.1010337)
Supplement: S1 Appendix — Supplementary Figures A-O and Tables A,B. Fig A in S1 Appendix. Power of 3P-CLR in the ancestral central-eastern population. Each ROC curve was generated from 1000 neutral simulations and 1000 selection simulations for each s (s = 0.05, 0.1). Fig B in S1 Appendix. Full unfolded SFS for the whole genome and each 3P-CLR tail threshold in centrals (top left), easterns (top right) and central-eastern combined (bottom). The central-eastern combined SFS was made by simply pooling all the samples as both subspecies have nearly identical sample sizes (central: 18, eastern: 19). The SFS are all indicative of selective sweeps. Fig C in S1 Appendix. Site frequency spectrum of SNPs in candidate windows. Allele frequencies of SNPs genome-wide and at different 3P-CLR tail thresholds. A: Unfolded SFS for central, eastern and central and eastern combined. The X axis is limited to focus on high-frequency derived alleles B: Absolute DAF difference between central-eastern and Nigeria-Cameroon. Fig D in S1 Appendix. Enrichment of gene ontology (GO) categories across candidate genes at different 3P-CLR quantiles in the central-eastern ancestor. Only categories with a significant enrichment in at least one quantile (FDR<0.05) are shown. Categories are separated by GO class: Biological Process, Cellular Component and Molecular Function. Colours represent FDR values (red as highest significance). Grey represents instances where a GO category was undetected in that particular quantile. Stars indicate a significant enrichment in that 3P-CLR quantile. Fig E in S1 Appendix. DAF of the three candidate SNPs of interest in CD4 across chimpanzee subspecies. These candidate SNPs correspond to those highlighted in Fig 4. SNP at chr12:6963043 represents a splice variant with signatures of positive selection in the central-eastern ancestor. SNPs at chr12:6978193 (V55I SNP) and chr12:6978232 (P68T SNP) are missense variants with signatures of positive selection in centrals and the central-eastern ances [file pgen.1010337.s001.docx]

# **Supplementary Material** **Genetic adaptations to SIV across chimpanzee populations**

**Supplementary Figures**


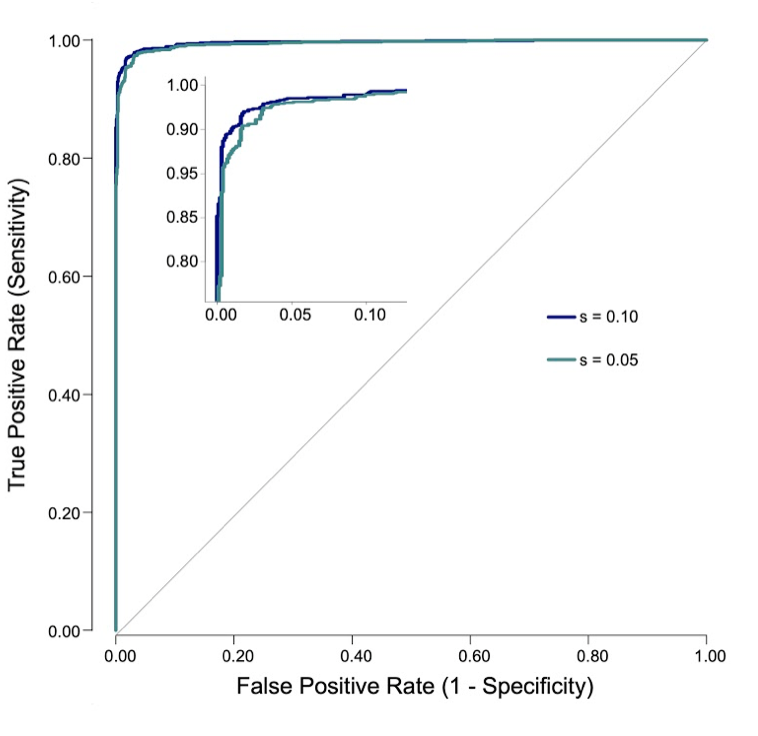


**Fig A:** **Power of 3P-CLR in the ancestral central-eastern population**. Each ROC curve was generated from 1000 neutral simulations and 1000 selection simulations for each s (s=0.05, 0.1).

####


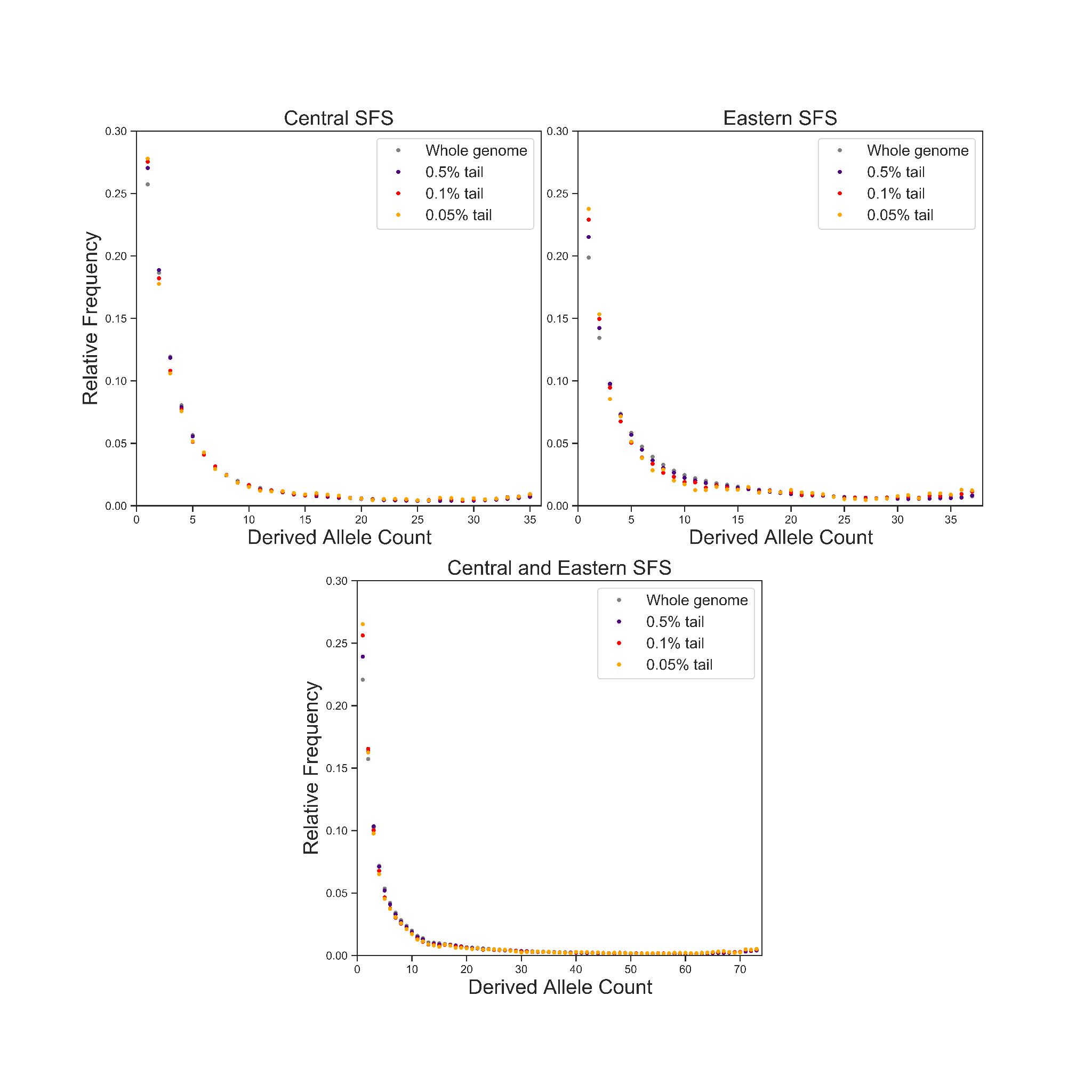


**Fig B: Full unfolded SFS for the whole genome and each 3P-CLR tail threshold in centrals (top left), easterns (top right) and central-eastern combined (bottom).** The central-eastern combined SFS was made by simply pooling all the samples as both subspecies have nearly identical sample sizes (central: 18, eastern: 19). The SFS are all indicative of selective sweeps.


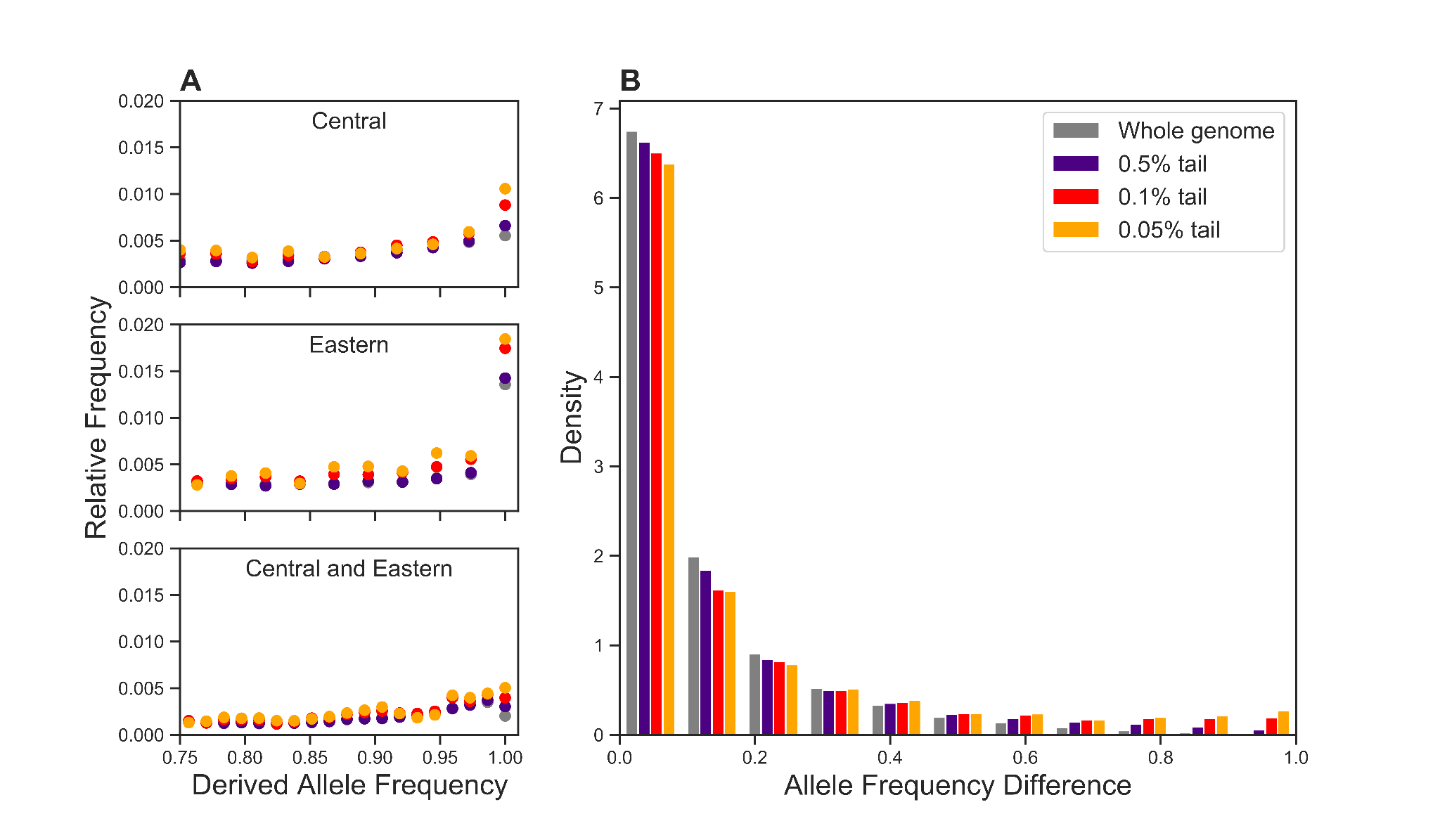


#### Fig C. Site frequency spectrum of SNPs in candidate windows. Allele frequencies of SNPs genome-wide and at different 3P-CLR tail thresholds. A: Unfolded SFS for central, eastern and central and eastern combined. The X axis is limited to focus on high-frequency derived alleles B: Absolute DAF difference between central-eastern and Nigeria-Cameroon.


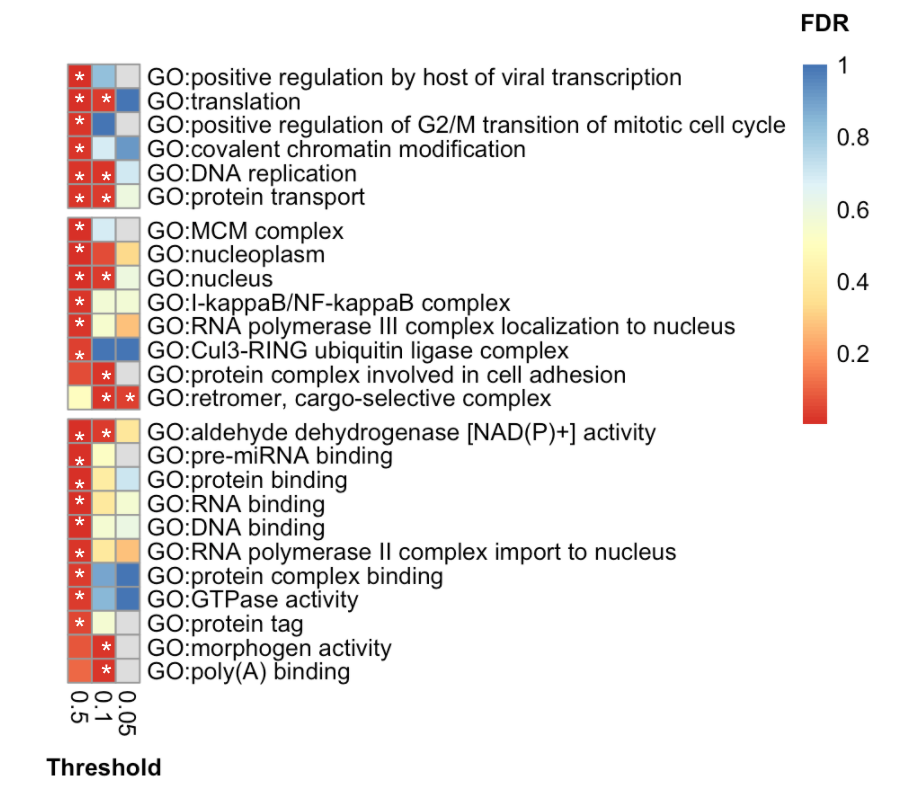


**Fig D: Enrichment of gene ontology (GO) categories across candidate genes at different 3P-CLR quantiles in the central-eastern ancestor**. Only categories with a significant enrichment in at least one quantile (FDR<0.05) are shown. Categories are separated by GO class: Biological Process, Cellular Component and Molecular Function. Colours represent FDR values (red as highest significance). Grey represents instances where a GO category was undetected in that particular quantile. Stars indicate a significant enrichment in that 3P-CLR quantile.


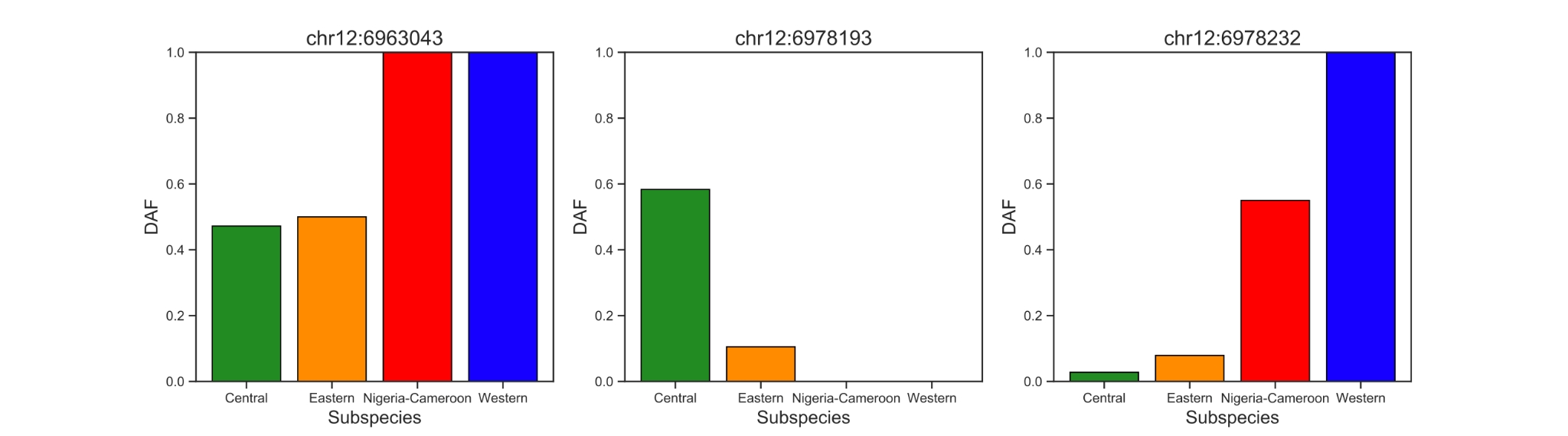


**Fig E: DAF of the three candidate SNPs of interest in *CD4* across chimpanzee subspecies.** These candidate SNPs correspond to those highlighted in Fig 4. SNP at chr12:6963043 represents a splice variant with signatures of positive selection in the central-eastern ancestor. SNPs at chr12:6978193 (V55I SNP) and chr12:6978232 (P68T SNP) are missense variants with signatures of positive selection in centrals and the central-eastern ancestor respectively.


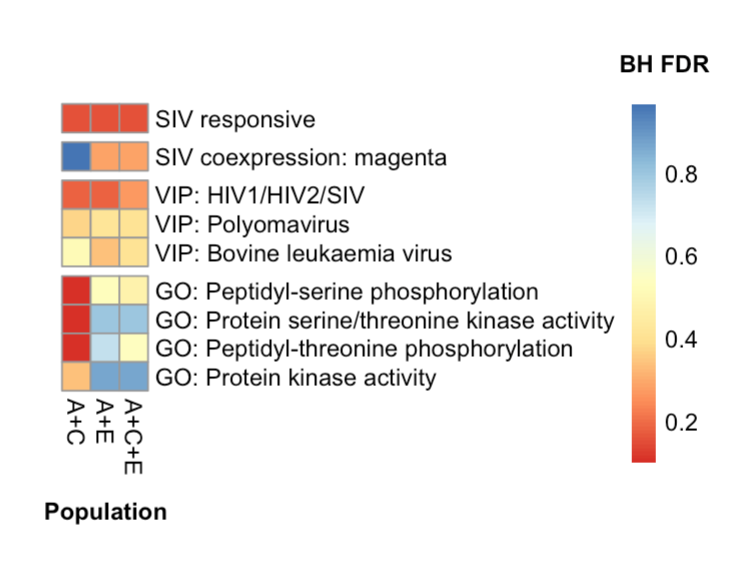


**Fig F: Enrichment in SIV-related, VIP and GO categories of candidate targets of positive selection across populations, tested using Gowinda**. Abbreviations indicate the populations tested: central-eastern ancestor + central (A+C), central-eastern ancestor + eastern (A+E), central-eastern ancestor + central + eastern (A+C+E). Categories are separated by gene set tested: SIV responsive genes, SIV co-expression modules, VIPs and GO categories [26,39,40,46-48]. Colours represent Benjamini and Hochberg corrected-FDR values (red as highest significance). We note that no category reaches significant enrichment (after correcting FDR values for the number of populations tested using a BH correction).


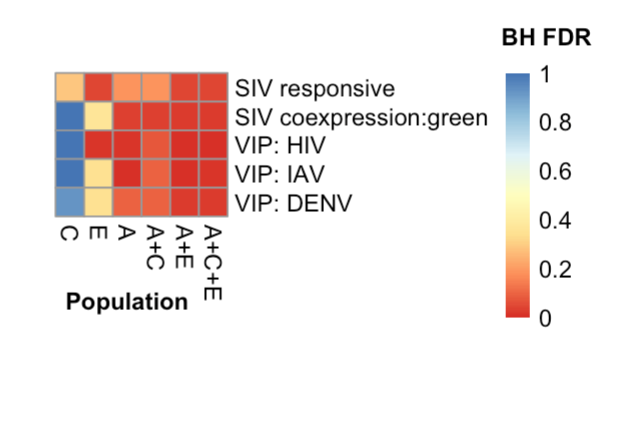


**Fig G: Enrichment in SIV-related, and VIP categories of candidate targets of positive selection across populations, tested using *set_perm*.** Abbreviations indicate the populations tested: central (C), eastern (E), central-eastern ancestor (A), central-eastern ancestor + central (A+C), central-eastern ancestor + eastern (A+E), central-eastern ancestor + central + eastern (A+C+E). The following categories are significantly enriched in the three populations together at BH-corrected FDR < 0.05: SIV responsive genes, SIV co-expression green module, HIV/SIV VIPs, influenza (IAV) VIPs and dengue (DENV) VIPs .All of these categories are also significantly enriched when combining the central-eastern ancestor and the eastern subspecies. The SIV co-expression green module is significantly enriched when combining the central-eastern ancestor and the central subspecies. In the single lineages, we see significant enrichment in the eastern subspecies in the SIV-responsive and HIV/SIV VIPs . While the central-eastern ancestor is significantly enriched in the SIV co-expression green module, HIV/SIV VIPs and influenza (IAV) VIPs .


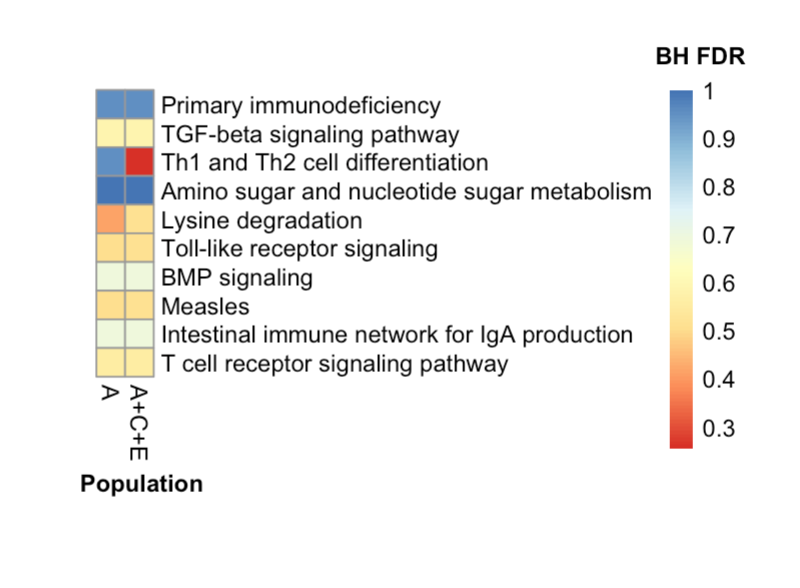


**Fig H:** **Enrichment in KEGG pathways of candidate targets of positive selection across populations, tested using Gowinda**. Abbreviations indicate the population(s) tested: central-eastern ancestor (A), central-eastern ancestor + central + eastern (A+C+E). For the central-eastern ancestor we used candidate genes in the least stringent quantile (0.5), to match the number of candidates for the subspecies. Colours represent BH-corrected FDR values (red as highest significance). We note that none of the KEGG pathways reaches BH-corrected FDR < 0.05. The strongest enrichment is for the Th1 and Th2 cell differentiation pathway for A+C+E with p-value=0.00056 and BH-FDR=0.25552, which represents a nominal enrichment.


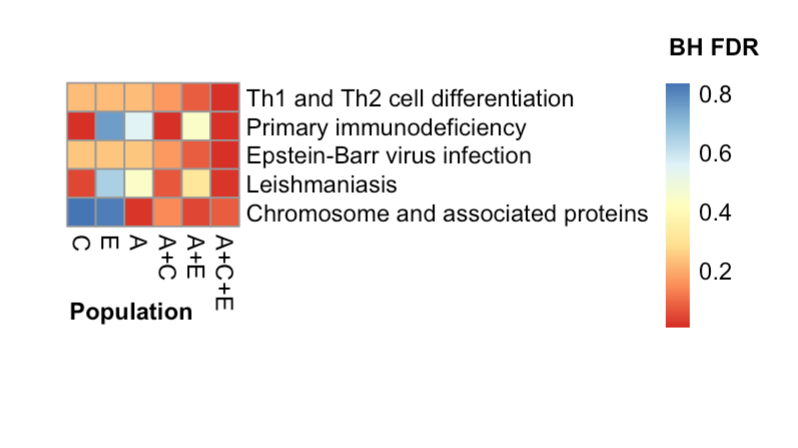


**Fig I:** **Enrichment in KEGG pathways of candidate targets of positive selection across populations, tested using *set_perm*.** Abbreviations indicate the populations tested: central-eastern ancestor + central (A+C), central-eastern ancestor + eastern (A+E), central-eastern ancestor + central + eastern (A+C+E). The following KEGG categories are significantly enriched in the three populations together at BH-corrected FDR < 0.05: 'Th1 and Th2 cell differentiation', 'primary immunodeficiency', 'Epstein-Barr virus infection' and 'leishmaniasis'. The 'primary immunodeficiency' and 'leishmaniasis' categories are also significantly enriched in the central subspecies. Hence for the 'primary immunodeficiency' and 'leishmaniasis' gene sets, the central lineage is likely driving the signal when the three populations are combined. When the central-eastern ancestor and eastern subspecies are combined, we see significant enrichment in the ‘chromosome and associated proteins’ pathway, which is also significantly enriched in the central-eastern ancestor.


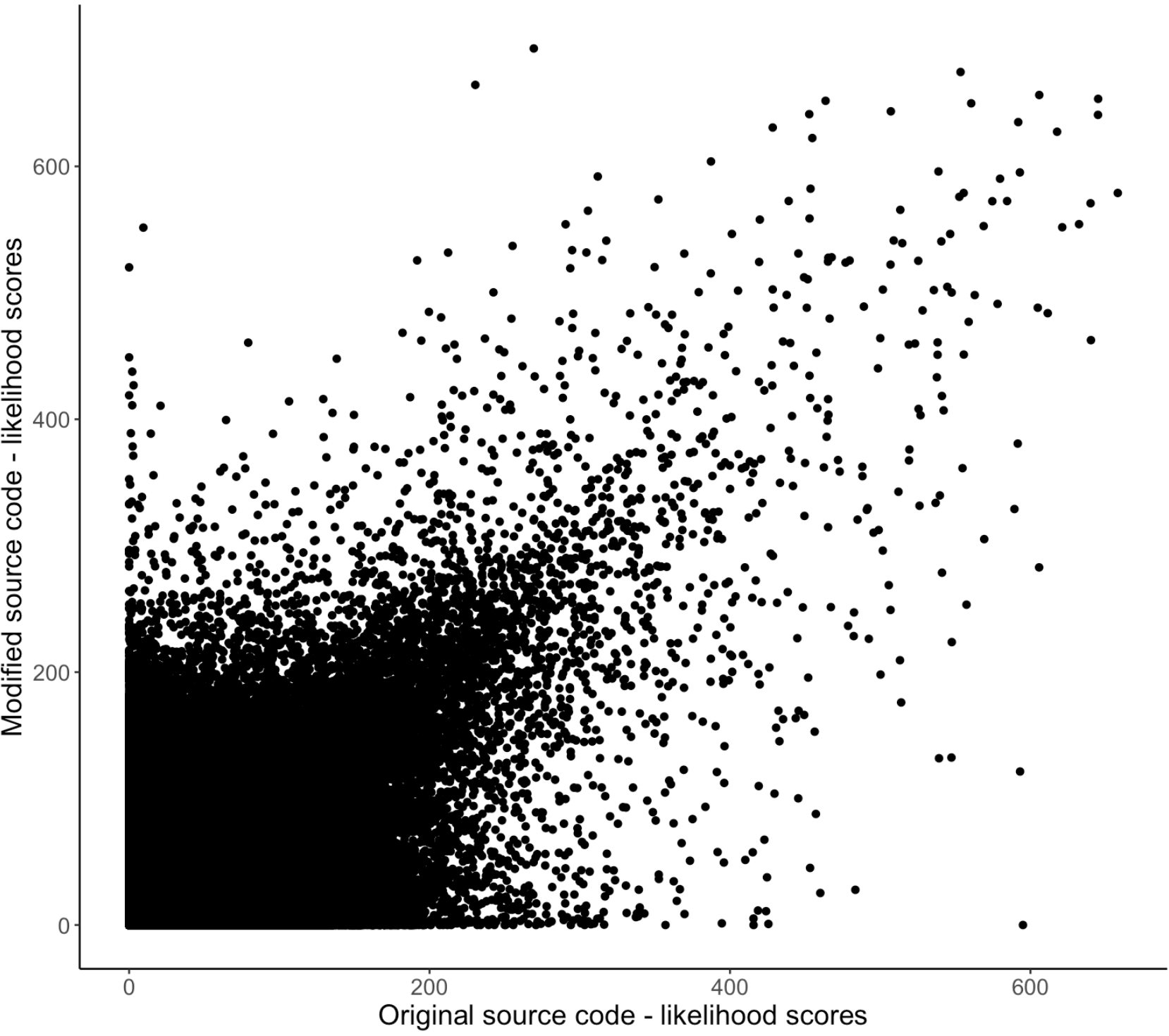


**Fig J: Correlation between the 3P-CLR values with the original and extended code.** Likelihood scores are significantly correlated between the original and modified 3P-CLR source code (rho=0.4565, p<2.2e-16). The absence of a perfect correlation is not due to differences in the algorithm, but due to the sampling variance of SNPs in each window. Specifically, if more than 100 SNPs are present within a given window, 3P-CLR chooses 100 SNPs at random. Thus, the same SNPs will not be sampled each time the method is run. Variation in 3P-CLR likelihood ratio scores therefore results if different SNPs were used to calculate the statistic. As expected, the correlation is weaker for low 3P-CLR but high at higher 3P-CLR values, where candidates of positive selection fall.


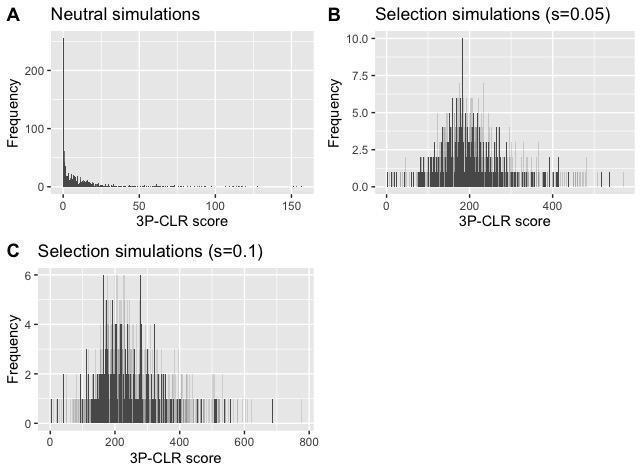


**Fig K: Distribution of 3P-CLR scores** for data simulated under neutrality (A) and positive selection with selection coefficients of 0.05 (B) and 0.1 (C), 1000 replicates were generated in each case.


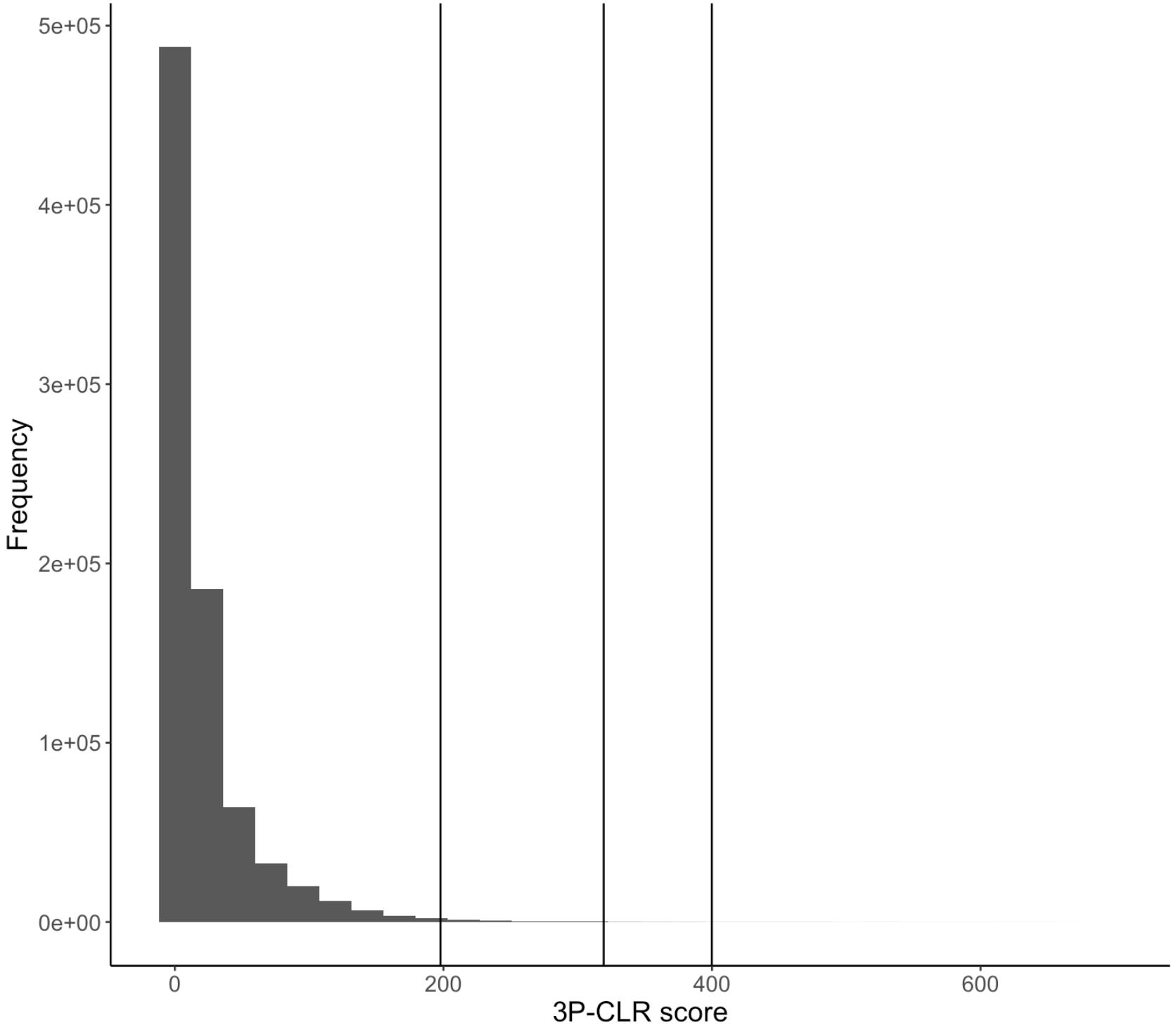


**Fig L:** **Empirical distribution of 3P-CLR scores**. Vertical lines indicate the thresholds used to define candidate windows (0.5%, 0.1% and 0.05% tails of the empirical distribution).


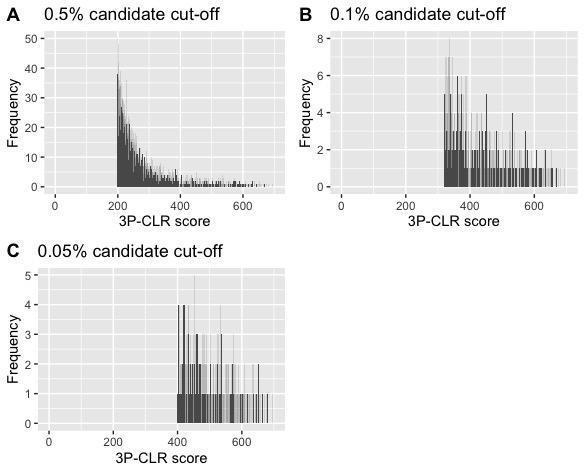


**Fig M:** **Distribution of 3P-CLR scores in the tails of the empirical distribution.** (A) the 0.5%, (B) 0.1% and (C) 0.05% candidate quantiles, corresponding to 4090, 818 and 409 genomic windows respectively.


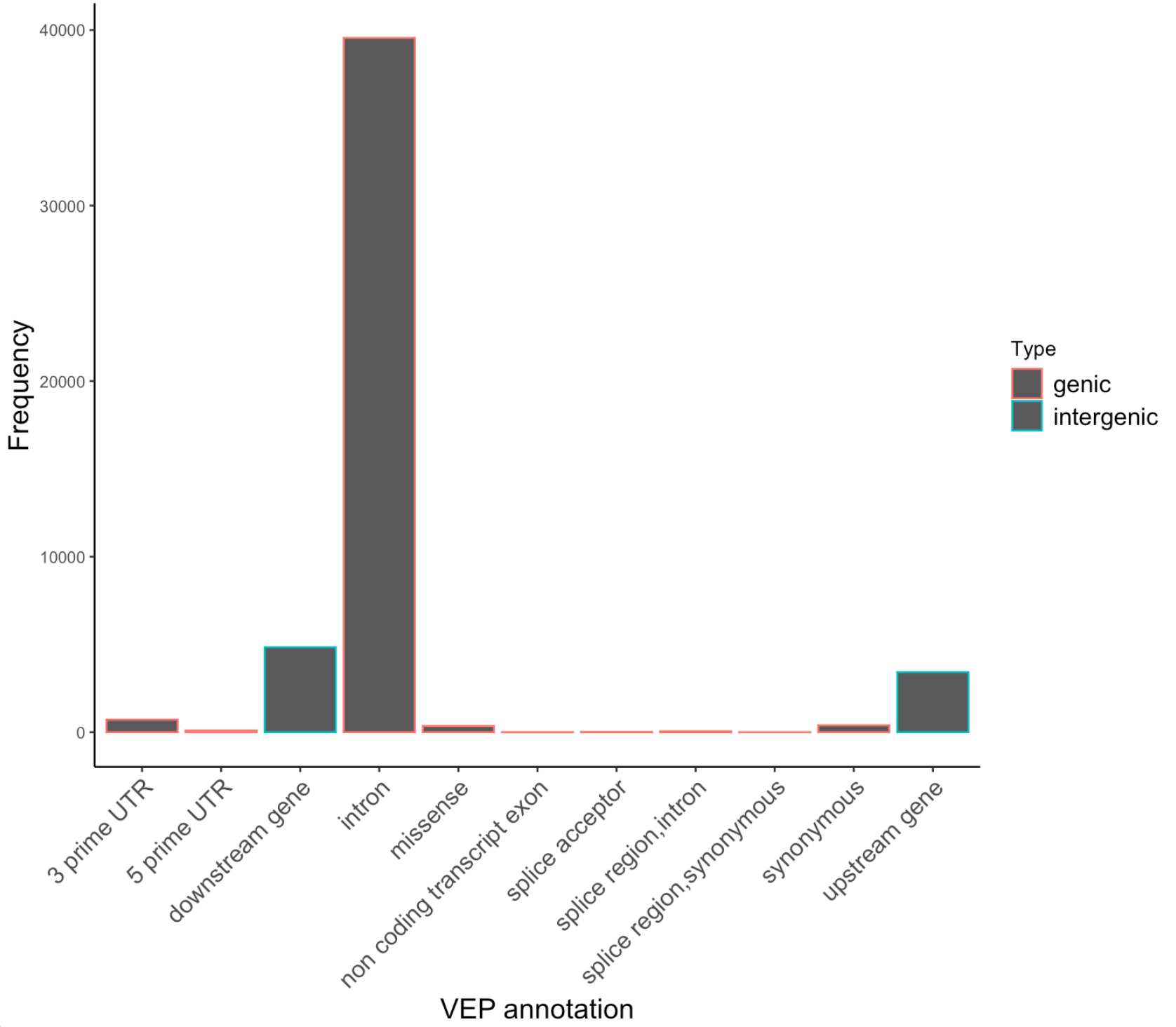


**Fig N: Functional annotation of 20 highest PBSnj SNPs per window at the 0.5% threshold using VEP**.


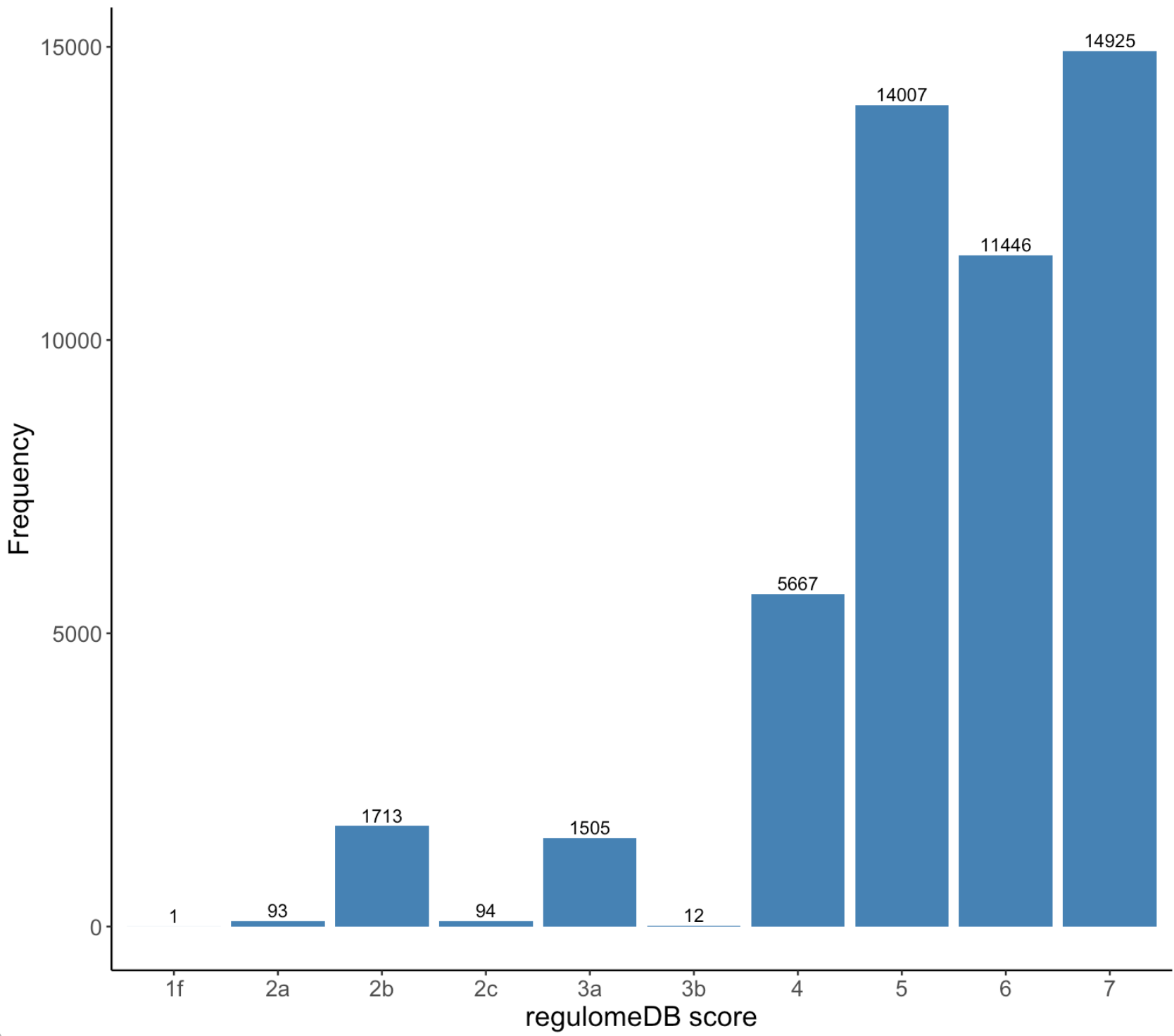


**Fig O:** **Functional annotation of 20 highest PBSnj SNPs per window at the 0.5% threshold using regulomeDB**.

**Supplementary Tables**


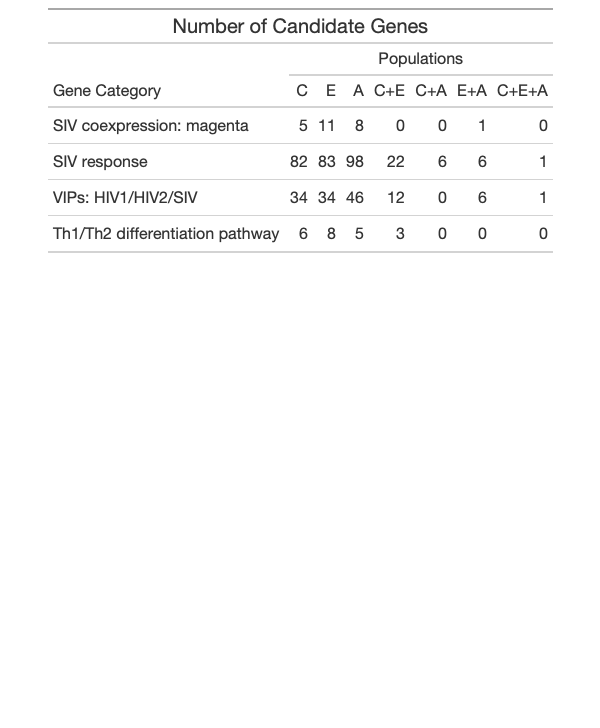


**Table A:** **Number of candidate genes from each population which belong to gene categories of interest.** The number of genes which are candidates in central only, eastern only, and ancestral only are shown in the C, E and A columns respectively. The number of genes which are candidates in both central and eastern, central and ancestral, eastern and ancestral, or all three populations are shown in the C+E, C+A, E+A, and C+E+A columns respectively.

| **Category** | **Gene** | **Location PBSnj score Annotation RegulomeDB score** |
| --- | --- | --- |
| SIV-response | *SHCBP1* | 16 45562119 45562120 0.977169244867928 upstream_gene_variant 2b |
| SIV-response | *RAI1* | 17 38254584 38254585 0.979097158400808 intron_variant 2b |
| SIV co-expression: module green | *OGFR* | 20 60321593 60321594 0.540581803741123 intron_variant 2b |
| SIV co-expression: module green | *OGFR* | 20 60322673 60322674 0.515259538932669 intron_variant 2b |
| SIV co-expression: module green | *NUDT16L* | 16 4750729 4750730 0.833342835848134 3_prime_UTR_variant 2b |
| SIV co-expression: module green | *NUDT16L* | 16 4750809 4750810 0.815198703617106 3_prime_UTR_variant 2b |
| VIPs: Influenza A | *FLNC* | 7 130323113 130323114 0.626039709365860 synonymous_variant(atT/atC) 2b |
| VIPs: Influenza A | *FLNC* | 7 130323188 130323189 0.630704319466529 synonymous_variant(aaT/aaC) 2b |
| VIPs: Influenza A | *MOB2* | 11 1491986 1491987 0.979097158400808 downstream_gene_variant 2b |
| VIPs: Influenza A; HIV1/HIV2/SIV | *AKAP8L* | 19 15714425 15714426 0.919066385805049 intron_variant 2b |
| VIPs: Influenza A; HIV1/HIV2/SIV | *MYH10* | 17 47588373 47588374 0.869165983371999 intron_variant 2b |
| VIPs: Influenza A | *TNRC18* | 7 4030370 4030371 0.886915240089703 intron_variant 2b |
| VIPs: Influenza A | *TNRC18* | 7 4061251 4061252 0.782208048674908 intron_variant 2b |
| VIPs: Influenza A | *CTPS1* | 1 41298930 41298931 0.831830891496028 intron_variant 2b |
| VIPs: Influenza A | *NT5C2* | 10 102355543 102355544 0.794666914005296 intron_variant 2b |
| VIPs: Influenza A | *NT5C2* | 10 102355847 102355848 0.794666914005296 intron_variant 2a |
| VIPs: Influenza A | *HNRNPUL1* | 19 46464301 46464302 0.717458128517244 intron_variant 2b |
| VIPs: Influenza A | *HNRNPUL1* | 19 46469387 46469388 0.724719690820092 intron_variant 1f |
| VIPs: Influenza A; HIV1/HIV2/SIV | *PSMB7* | 9 123481626 123481627 0.762192773400031 upstream_gene_variant 2b |
| VIPs: Influenza A; HCMV | *RAB10* | 2A 26388071 26388072 0.969476652360052 5_prime_UTR_variant 2b |
| VIPs: HIV1/HIV2/SIV | *ITGAL* | 16 30634230 30634231 0.883503460161937 intron_variant 2b |
| VIPs: HIV1/HIV2/SIV | *SPATA5L1* | 15 42554379 42554380 0.833342835848134 intron_variant 2b |
| VIPs: HIV1/HIV2/SIV | *WHSC1* | 4 2021347 2021348 0.878045955284611 3_prime_UTR_variant 2b |
| VIPs: HIV1/HIV2/SIV | *WHSC1* | 4 2021461 2021462 0.919066385805049 3_prime_UTR_variant 2b |
| VIPs: HIV1/HIV2/SIV | *WHSC1* | 4 2021623 2021624 0.897971129460690 3_prime_UTR_variant 2b |
| VIPs: HIV1/HIV2/SIV | *GNAO1* | 16 55330504 55330505 0.639189125279027 intron_variant 2b |
| VIPs: HIV1/HIV2/SIV | *SPATS2* | 12 39874385 39874386 0.823158026244118 intron_variant 2b |
| VIPs: HIV1/HIV2/SIV | *SMG6* | 17 2083071 2083072 0.904042285992019 intron_variant 2a |
| VIPs: HIV1/HIV2/SIV | *SMG6* | 17 2083141 2083142 0.904042285992019 intron_variant 2b |
| VIPs: HIV1/HIV2/SIV | *SMG6* | 17 2083347 2083348 0.904042285992019 intron_variant 2b |
| VIPs: HIV1/HIV2/SIV | *RAD23A* | 19 13216455 13216456 0.958872156258472 upstream_gene_variant 2b |
| VIPs: HIV1/HIV2/SIV | *CERS2* | 1 129153708 129153709 0.777225715621888 intron_variant 2b |
| VIPs: HCMV | *USP54* | 10 72098548 72098549 0.477321510964906 missense_variant (T/A Aca/Gca) - |
| VIPs: ADV | *RAB34* | 17 28361600 28361601 0.712341200918947 upstream_gene_variant 2b |
| VIPs: ADV | *RAB34* | 17 28361637 28361638 0.698462506757592 upstream_gene_variant 2b |
| VIPs: ADV | *RAB34* | 17 28362090 28362091 0.724251911659941 synonymous_variant(ccG/ccC) 2b |

**Table B: SNPs of interest in genes identified within candidate windows of positive selection at the 0.5% threshold in the central-eastern ancestor, additional to *CD4.*** SNPs with regulomeDB scores of 1f, 2a, 2b, 2c are considered to have putative significant regulatory function, due to the lack of eQTL data for chimpanzees. Category indicates the gene set each gene belongs to. Abbreviations: HCMV and ADV indicate human cytomegalovirus and adenovirus respectively.
